# Ferroptosis: Newly Emerged Regulator for Human Disease

**DOI:** 10.1002/mco2.70921

**Published:** 2026-08-19

**Authors:** Dandan Xiao, Lin Ye, Tianqi Jiang, Mengyuan Liu, Yuxia Zhou

**Affiliations:** ^1^ Department of Clinical Laboratory Shandong Provincial Maternal and Child Health Care Hospital Affiliated to Qingdao University Jinan China; ^2^ Institute for Translational Medicine Qingdao University Qingdao China

**Keywords:** acute organ injury, cancer, cardiovascular diseases, ferroptosis, molecular regulation, neurodegenerative disorders

## Abstract

Ferroptosis is a novel, iron‐driven form of cell death characterized by iron‐dependent membrane‐lipid peroxidation. Accumulating evidence has revealed that ferroptosis is heavily involved in multiple physiological and pathological conditions, resulting in remarkable progress in the treatment of multiple diseases. This review summarizes recent advances in and the fundamental features of ferroptosis and outlines the molecular mechanisms that regulate core metabolic pathways and their relevance to cellular physiology. In addition, the mechanisms and functional implications of ferroptosis in cancer, cardiovascular diseases, neurodegenerative disorders, and acute organ injury are discussed, and current diagnostic and therapeutic strategies that target ferroptosis are summarized. Finally, the review identifies persistent gaps in the mechanistic understanding, relevant biomarker development, and translational validity of ferroptosis to provide a comprehensive foundation for future research and clinical innovation in the era of precision medicine.

## Graphical abstract

Ferroptosis is a key regulated cell death pathway closely involved in cancers, neurodegenerative disorders, acute organ injuries, and cardiovascular and chronic metabolic diseases, representing a promising therapeutic target for diverse human illnesses. (Graphical abstract was drawn using the BioGDP platform.)

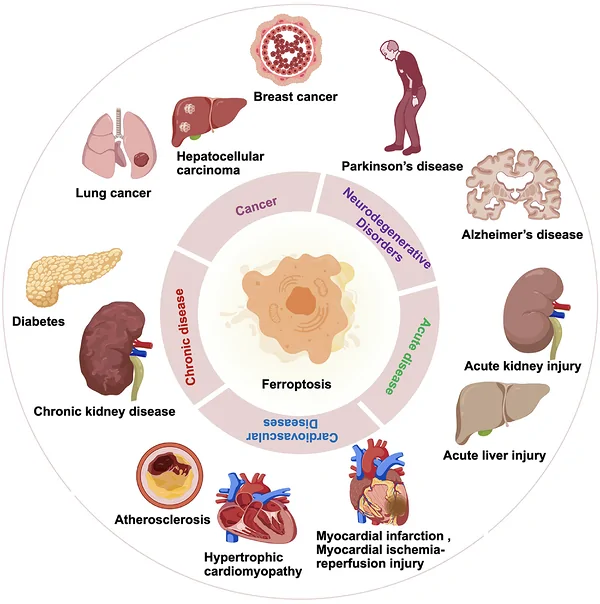

## 1 Introduction

Cell death is a fundamental biological process that underlies tissue maturation and organismal homeostasis. Over the past decades, research has uncovered a diverse landscape of modalities responsible for regulated cell death (RCD). Following the landmark identification of apoptosis [1], and the subsequent discoveries of pyroptosis [2], necroptosis [3], cuproptosis [4], and others—ferroptosis has emerged as a distinct and biologically significant RCD modality [5]. Defined in 2012 as an iron‐dependent, nonapoptotic form of cell death driven by lethal lipid peroxidation, today, ferroptosis is recognized as a key player in cancer, cardiovascular diseases, neurodegenerative disorders (NDDs), and acute organ injury. Its unique reliance on iron metabolism, lipid peroxidation, and failure of the glutathione–glutathione peroxidase 4 (GPX4) antioxidant axis failure distinguishes it from other RCD pathways and makes it a critical node in both physiological and disease processes.

**FIGURE 1:**
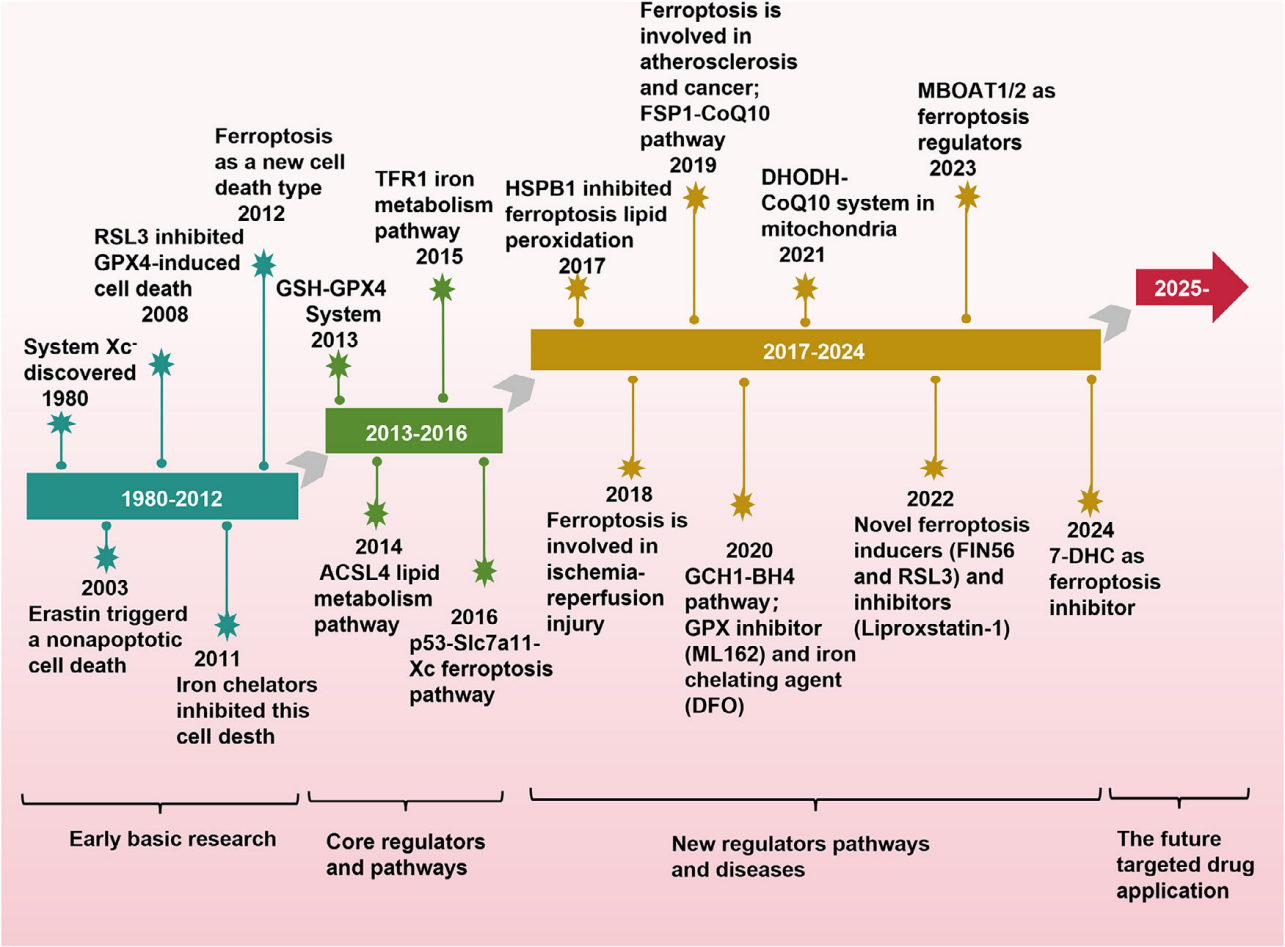
History of research on the discovery and development of ferroptosis. The term “ferroptosis” was officially coined in 2012; the foundational understanding of this process can be traced back to studies conducted prior to 1980. Since its formal identification in 2012, research into ferroptosis and its regulatory mechanisms has significantly expanded and flourished. RSL3, RAS‐selective lethal compound 3; GPX4, glutathione peroxidase 4; GSH, glutathione; TFR1, transferrin receptor 1; ACSL4, acyl‐CoA synthetase long chain family member 4; HSPB1, heat shock protein family B member 1; FSP1, ferroptosis suppressor protein 1; BH4, tetrahydrobiopterin; CoQ_10_, coenzyme Q_10_; GCH1, GTP cyclohydrolase 1; DHODH, dihydroorotate dehydrogenase; MBOAT1/2, membrane‐bound O‐acyltransferase domain‐containing 1/2; 7‐DHC, 7‐dehydrocholesterol.

The identification of ferroptosis followed from earlier observations of nonapoptotic cell death: erastin was first identified in 2003 as an inducer of tumor cell death independent of apoptosis [6], followed by the discovery of Ras‐selective lethal compounds (RSL3/RSL5) in 2008, which trigger an iron‐dependent, oxidative cell death that can be inhibited by iron chelators and antioxidants [7]. These findings culminated in the assignment of the name “ferroptosis” in 2012 [5], defined as an iron‐dependent, nonapoptotic RCD distinct from apoptosis, necrosis, and autophagy [8] (see Figure 1). Morphologically, ferroptosis is characterized by cell membrane instability, cytoskeletal rearrangement, and mitochondrial alterations (including increased membrane density, cristae reduction, and outer membrane rupture) [9]. Biochemically, ferroptosis is characterized by glutathione depletion, impaired GPX4 activity, iron accumulation, lipid peroxidation, and increased reactive oxygen species (ROS) [10], which occur primarily through core mechanisms including iron metabolism imbalance, lipid peroxidation chain reactions, and antioxidant system dysregulation [11].

Unlike traditional forms of cell death such as apoptosis and necrosis, ferroptosis modulates glutathione metabolism, iron homeostasis, and lipid‐remodeling pathways to play pivotal roles in cancer, NDDs, cardiovascular disorders, and organ injury [12, 13, 14, 15]. These unique mechanisms give ferroptosis‐targeted therapy two forms of clinical potential: inducing ferroptosis can eliminate malignant cells and overcome drug resistance in tumors, whereas suppressing it protects vital cells in degenerative or injury‐related pathologies. Over the past decade, high‐throughput screening, gene editing, and lipidomics have helped refine the ferroptosis regulatory map and revealed the promise of this form of cell death for treating incurable noncommunicable diseases, elevating it from a basic scientific concept to a potential translational target in medicine.

Despite these advances, critical bottlenecks in this translation persist—including an incomplete understanding of the tissue‐specific regulation of ferroptosis, challenges in distinguishing ferroptosis from other methods of RCD in complex pathological contexts, and limitations in translating preclinical findings to clinical practice. This review summarizes recent progress in the understanding of ferroptosis regulatory mechanisms, with a focus on the dynamic relationship among iron metabolism, lipid peroxidation, and antioxidant defense, as well as crosstalk with other RCD pathways, to provide a basis for understanding coordinated cell fate control. We further discuss the role of ferroptosis in human diseases and critically evaluate major limitations in existing detection and therapeutic‐targeting approaches. On the basis of these emerging findings, we propose future research directions for advancing both basic and translational studies in this rapidly evolving field.

## 2 The Core Metabolic Circuitry of Ferroptosis

Ferroptosis is a unique, iron‐dependent form of RCD. Unlike traditional forms of cell death, ferroptosis is governed by a complex metabolic network based primarily on iron metabolism, lipid peroxidation, and antioxidant defense axes whose interdependencies define the cell‐death process: iron metabolism acts as the driver, polyunsaturated fatty acid‐containing lipids act as the fuel, and antioxidant defense acts as the protective barrier. In this way, ferroptosis is jointly determined by iron‐driven lipid peroxidation and the collapse of antioxidant defenses through the dynamic interplay among these three axes. This review outlines the molecular mechanisms underlying these pathways and interactions of ferroptosis and other cell death modes, aiming to inform the development of relevant therapeutic strategies.

### 2.1 The Driver: Dysregulated Iron Metabolism and Redox‐Active Labile Iron Pool

As the upstream, core node that drives the entire ferroptosis metabolic network, iron metabolism is a prerequisite basis for lipid peroxidation and subsequent ferroptosis, forming the functional connection between the substrate lipid axis and the antioxidant defense axis. Iron is a critical cofactor supports the catalytic activity of various metabolic enzymes involved in the production of ROS, notably lipoxygenases (LOXs) and cytochrome P450 oxidoreductase (POR). Moreover, iron fuels nonenzymatic Fenton chemistry, in which phospholipid hydroperoxides (PLOOHs) react with Fe^2+^ or Fe^3+^ to generate lipid radicals (PLO• and PLOO•), which in turn, propagate lipid peroxidation chains by oxidizing polyunsaturated fatty‐acid phospholipids (PUFA‐PLs), thereby increasing PLOOH accumulation, compromising membrane integrity, and triggering ferroptosis. This downstream effector mechanism directly defines the dominant driving role of iron metabolism in the initiation of ferroptosis.

Intracellular iron homeostasis, the core process through which iron exerts its regulatory role, is governed strictly by core functional nodes (import, storage, and export) and their key associated regulatory factors. Heme iron is directly absorbed by heme carrier protein 1 (HCP1) at the apices of intestinal epithelial cells and then catalyzed by hemeoxygenase‐1 (HO‐1) [16], while nonheme Fe^3+^ is transported by divalent metal transporter 1 (DMT1) [17]. Fe^2+^ can be stored in ferritin or released into the circulation through ferroportin (FPN), the only known protein that exports nonheme iron out of cells [18], while iron is distributed to peripheral tissues via transferrin receptor 1 (TFR1)‐mediated internalization [19]. Among these factors, TF/TFR1, FPN, and ferritin are core regulatory nodes for general iron flux, whereas auxiliary factors such as HCP1 and DMT1 facilitate specific iron transport processes.

On the basis of these core nodes, several key regulatory factors directly modulate ferroptosis sensitivity by altering iron homeostasis. TF and its receptor TFRC jointly promote ferroptosis through the import of iron into cells; in the absence of TF, solute carrier family 39 member 14 (SLC39A14) is consequently upregulated in hepatocytes, leading to excessive iron import. Conversely, inhibiting iron import or potentiating cellular iron export confers ferroptosis resistance, as evidenced by the ability of iron chelators such as deferoxamine (DFO) to block ferroptosis. In addition to iron transporters, iron storage regulators play critical roles. For example, receptor coactivator 4 (NCOA4)‐mediated ferritinophagy releases iron to increase ferroptosis sensitivity [20], whereas ferritin heavy chain 1 (FTH1) deletion enhances ferroptosis in vivo [21]. These findings collectively illustrate how core regulators of iron import, export, and storage are central to modulating ferroptosis.

**FIGURE 2:**
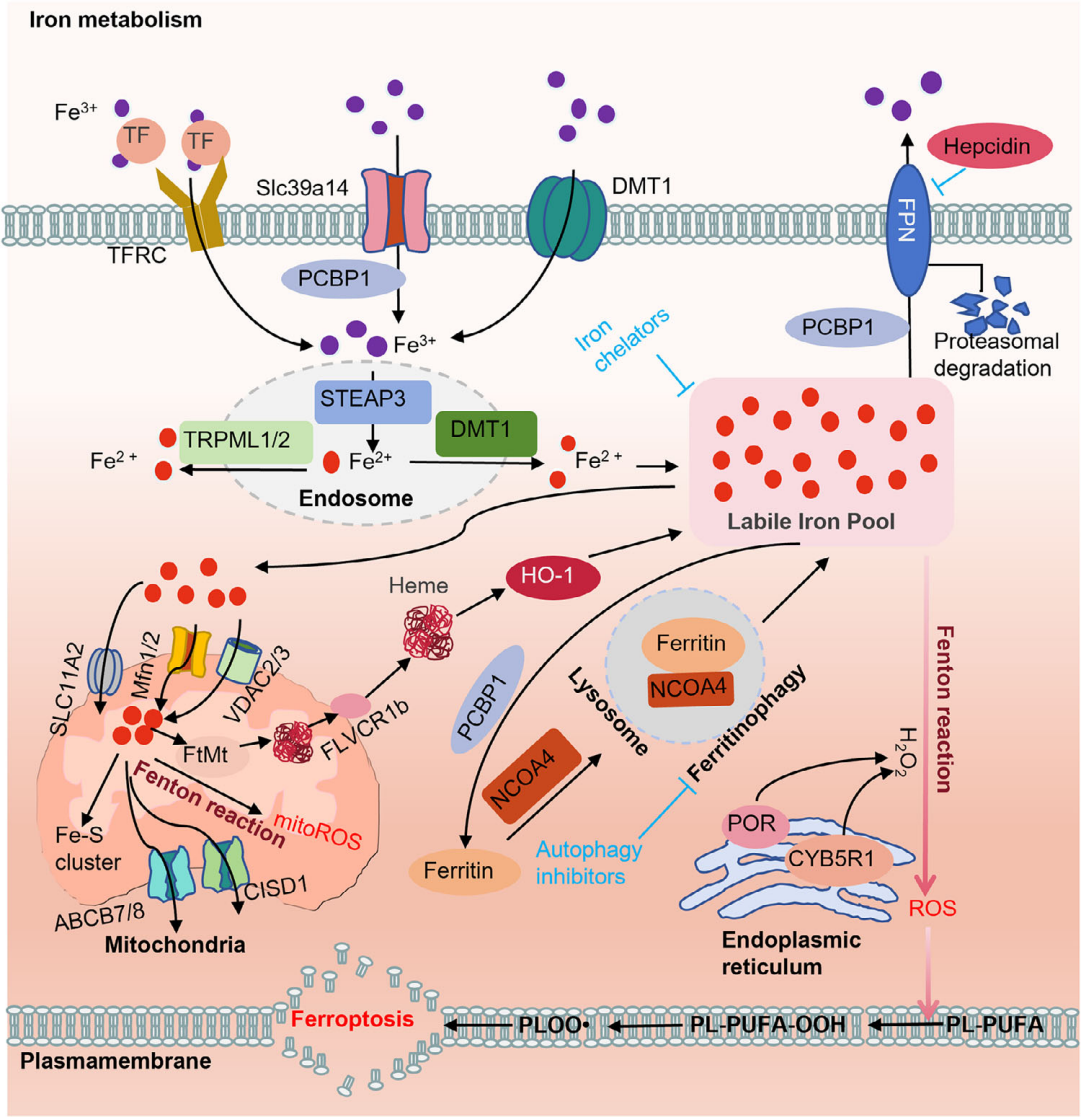
Targeting ferroptosis in iron metabolism. Ferroptosis is a regulated cell death process that is closely associated with iron metabolic pathways, encompassing the absorption, transportation, storage, and utilization of iron. In cytoplasmic iron metabolism, nonheme iron enters the cytoplasm via transporters such as TFRC, SLC39A14, and DMT1, subsequently being internalized into endosomes. Within endosomes, Fe^3+^ is reduced to Fe^2+^ by STEAP3, and Fe^2+^ is then released into the cytoplasm through DMT1. A portion of Fe^2+^ is stored in the LIP or sequestered within ferritin, while another fraction is exported out of the cell via FPN. Hepcidin promotes the internalization of FPN and its subsequent lysosomal degradation, thereby decreasing iron export. Ferritin can undergo selective ferritinophagy degradation mediated by its receptor NCOA4, thereby releasing Fe^2+^. Through the Fenton reaction, Fe^2+^ generates ROS, leading to lipid peroxidation in cell membranes and ultimately inducing ferroptosis. In mitochondrial iron metabolism, cytoplasmic Fe^2+^ enters mitochondria via transporters such as SLC11A2, Mfn1/2, and VDAC2/3. Inside mitochondria, Fe^2+^ contributes to the synthesis of iron–sulfur (Fe–S) clusters and is stored in FtMt. Excess Fe^2+^ can be transported back to the cytoplasm via ABCB7/8 and CISD1. FtMt plays a role in heme biosynthesis, and newly synthesized heme is exported from mitochondria via FLVCR1. Additionally, Fe^2+^ participates in the Fenton reaction within mitochondria, generating mitoROS. Furthermore, proteins such as POR and CYB5R1 in the endoplasmic reticulum contribute to the Fenton reaction, promoting ferroptosis. TFR1, transferrin receptor 1; TF, transferrin; DMT1, divalent metal transporter 1; STEAP3, six‐transmembrane epithelial antigen of prostate 3; PCBP1, poly(RC) binding protein 1; LIP, labile iron pool; ROS, reactive oxygen species; FPN, ferroportin; Mfrn1/2, mitoferrin 1/2; VDAC2/3, voltage‐dependent anion‐selective channel 2/3; ABCB7/8, ATP‐binding cassette transporter 7/8; mitoROS, mitochondrial reactive oxygen species; FtMt, mitochondrial ferritin; CISD1, CDGSH iron sulfur domain 1; FLVCR1, feline leukemia virus subgroup C cellular receptor 1; TRPML, lysosomal cation channel mucolipin; SLC39A14, solute carrier family 39 member 14; SLC11A2, solute carrier family 11 member 2; NCOA4, nuclear receptor coactivator 4; CYB5R1, cytochrome b5 reductase 1, POR, cytochrome P450 reductase; HO‐1, hemeoxygenase‐1.

In addition to cytoplasmic iron homeostasis, mitochondrial iron homeostasis also plays an important role in preventing ferroptosis. A specialized layer in the regulation of iron, mitochondrial iron handling involves import, storage, and export factors. Iron enters the mitochondrial matrix through solute carrier family 11 member 2 (SLC11A2), SLC25A37, and SLC25A28 forbiosynthesizing heme and Fe/S clusters [22], a process in which SLC25A37 and SLC25A28 act as key mitochondrial iron importers: SLC25A28 deletion inhibits erastin‐induced ferroptosis, while its overexpression promotes it [23]. Mitochondrial iron storage and export factors alter ferroptosis sensitivity. Ferritin mitochondrial (FTMT) sequesters iron to inhibit ferroptosis [24], whereas ATP binding cassette subfamily B member 8 (ABCB8) promotes mitochondrial iron export to reduce ferroptosis [25], unlike ABCB7, which does not directly affect ferroptosis despite its role in regulating mitochondrial iron transfer [26]. Notably, CDGSH iron sulfur domain 1 (CISD1) acts as a critical modulator by binding to VDAC to regulate mitochondrial iron import—CISD1, knockdown increases mitochondrial Fe^2+^ content and ferroptosis [27, 28], whereas HO‐1 activation induces mitochondrial iron overload to enhance ferroptosis in vitro and in vivo [29]. Furthermore, POR in the endoplasmic reticulum contributes to contribute to the initiation of lipid peroxidation during ferroptosis [30]. In summary, these results provide strong evidence that controlling the regulation of iron metabolism regulation affects sensitivity to ferroptosis and the specific results are shown in Figure 2.

### 2.2 The Fuel: Peroxidation of PUFAs in Membrane Phospholipids

Lipid molecules not only are the constitute fundamental structural components of cellular membranes but also play multiple roles in energy metabolism and cellular signal transduction [31]. A key event in ferroptosis is the enzymatic oxidation of PUFA‐PLs within cellular membranes represents a pivotal event in ferroptosis. At the core of ferroptosis lies three interrelated axes: lipid peroxidation substrates (PUFA‐PLs), iron as a catalytic mediator, and the GPX4‐mediated detoxification system. Iron functions as an essential catalytic mediator of the subsequent oxidative cascade: when nascent phospholipid hydroperoxides (PLOOHs) are not efficiently detoxified by GPX4, Fe^2^^+^ promotes their homolytic decomposition into alkoxyl and peroxyl radicals, thereby propagating the lipid peroxidation chain reaction and further amplifying PLOOH formation and accumulation [32, 33, 34]. Oxidized lipids are specifically produced during ferroptosis and accumulate in the mitochondria, lysosomes, and endoplasmic reticulum, suggesting that subcellular organelles play roles in phospholipid peroxidation and the activation of ferroptosis signaling [35].

To sustain the core PUFA‐PL peroxidation axis, key and auxiliary regulatory factors responsible for ensuring the availability and synthesis of these phospholipids work in concert to modulate ferroptosis sensitivity. Cluster of differentiation 36 (CD36) and fatty acid transport protein (FATP) serve as upstream key upstream nodes by mediating the uptake of free fatty acids uptake into cells, providing essential substrates for membrane phospholipid biosynthesis [36]. Acyl‐CoA synthetase long chain family member 4 (ACSL4) and lysophosphatidylcholine acyltransferase 3 (LPCAT3) are additional key downstream key regulators of PUFA‐PL synthesis and critical drivers of ferroptosis [37]. Studies have reported that overexpression of ACSL4 overexpression increases the proportion of polyunsaturated fatty acid phospholipids among phospholipids, increasing susceptibility to ferroptosis [38]. In contrast, genetic ablation or pharmacological inhibition of ACSL4 triggers a sharp shift in the composition of phospholipids from long‐chain PUFA tails to short‐chain and monounsaturated fatty acids (MUFAs), resulting in the inhibition of ferroptosis. Similarly, exogenous MUFA supplementation, or the ACSL3‐catalyzed conversion of MUFAs to acyl‐CoA (which replaces the PUFAs in the phospholipids) reduces cellular sensitivity to ferroptosis [39]. Conversely, LPCAT3 catalyzes the insertion of acylated PUFAs into membrane phospholipids; the deletion of this enzyme decreases the PUFA‐PL content and increases cellular resistance to ferroptosis. LOX is another key enzyme in multiple lipid metabolism pathways. This nonheme iron‐dependent dioxygenase that directly oxidizes PUFAs and PUFA‐containing lipids in cell membranes, thereby activating ferroptosis [35]. However, the role of LOX in ferroptosis remains controversial. On the one hand, 15‐LOX, when bound to phosphatidylethanolamine‐binding protein 1 (PEBP1), specifically catalyzes the oxidation of arachidonic acid‐containing membrane phospholipids (PE‐AAs) to produce 15‐HpETE‐PE, a lipid peroxide widely recognized as a hallmark of ferroptosis [40]. Moreover, various members of the LOX family, including ALOXE3, ALOX5, and ALOX12B, have been implicated in ferroptosis induction [41]. On the other hand, 15‐LOX deletion cannot rescue the embryonic lethality in GPX4 knockout mice or prevent cell death in adult mice with systemic GPX4 ablation [42]; furthermore, some cell lines do not express any major LOX enzymes [43]. In summary, LOX may contribute to ferroptosis in specific contexts but is not universally essential to the process, and its exact role and underlying mechanisms require further investigation. Another auxiliary modulator, membrane‐bound glycerophospholipid o‐acyltransferase 1/2, a pair of PL‐modifying enzymes, were identified by Liang et al. as novel sex hormone‐dependent ferroptosis suppressors; mechanistically, MBOAT1/2 inhibit ferroptosis by remodeling the cellular PL composition [44].

**FIGURE 3:**
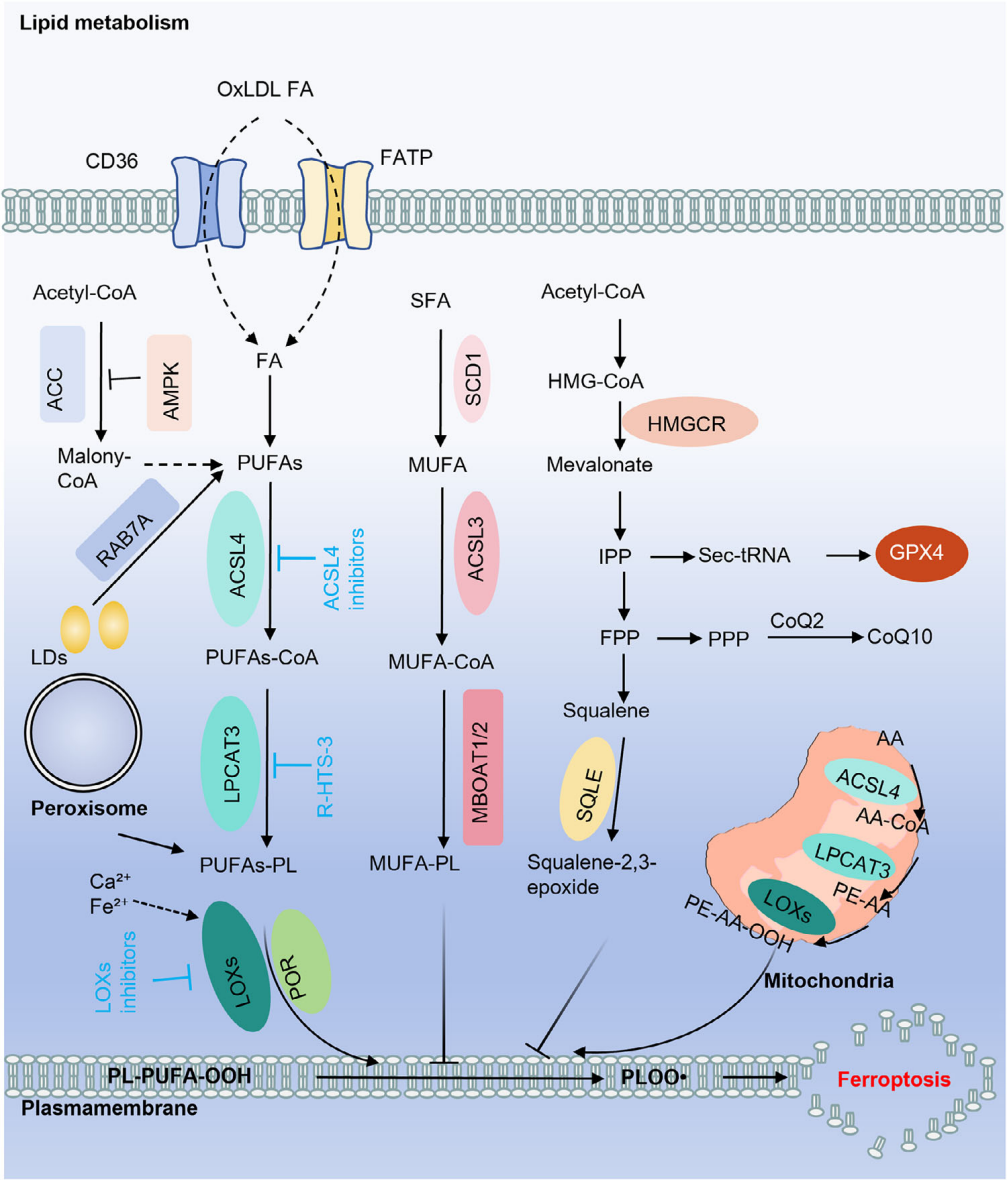
Targeting ferroptosis in lipid metabolism pathways. Ferroptosis is triggered by lipid peroxidation and involves the participation of multiple enzymes, including ACSLs, LPCAT3, ALOXs, and POR. This process is further modulated by fatty acid metabolism, encompassing PUFA uptake mediated by CD36 or FATP transporter proteins, ACC‐dependent PUFA synthesis, PUFA generation induced by lipid autophagy, and the mevalonate pathway‐driven synthesis of acetyl‐CoA for cholesterol, which contributes to the production or elongation of new fatty acids. ACC, acetyl‐CoA carboxylase; AA, arachidonic acid; CD36, cluster differentiation 36; FATP, fatty acid transport protein; CoQ2, coenzyme Q2;CoQ10, coenzyme Q10; CoA, acyl‐coenzyme A; ACSL 3/4, acyl‐CoA synthetase long chain family member 3/4; ALOXs, arachidonate lipoxygenase‐s; AMPK, AMP‐activated protein kinase; LPCAT3, lysophosphatidylcholine acyltransferase 3; PUFA, polyunsaturated fatty acid; PUFA‐PL, phospholipid‐containing polyunsaturated fatty acid; SQLE, squalene epoxidase; SFA, saturated fatty acids; MUFA, monounsaturated fatty acids; HMGCR, 3‐hydroxy‐3‐methylglutaryl‐CoA reductase; POR, cytochrome P450 oxidoreductase; IPP, isopentenyl pyrophosphate; GPX4, glutathione peroxidase 4; SCD1, stearoyl‐CoA desaturase1; MBOAT1/2, membrane‐bound O‐acyltransferase domain‐containing 1 and 2; LDs, lipid droplets; FPP, farnesyl pyrophosphate; PPP, pentose phosphate pathway.

Peroxisome‐mediated plasmalogen biosynthesis is considered another pathway that induces the lipid peroxidation of PUFA‐PLs, which favors the occurrence of ferroptosis [45]. RAB7A‐related autophagy‐induced selective degradation of lipid droplets increases the production of free fatty acids and promotes lipid peroxidation and subsequent ferroptosis [46]. In the cholesterol synthesis pathway, the downregulation of squalene monooxygenase, which catalyzes the oxidation of squalene to 2,3‐oxidosqualene, induces ferroptosis resistance in certain cancer cells [47]. Isopentenyl pyrophosphate, an intermediate in the cholesterol synthesis pathway, regulates selenocysteine tRNA, thus affecting GPX4 synthesis [48]. The inhibition of the mevalonate pathway reduces CoQ10 synthesis and disrupts ferroptosis signaling. These findings suggest that cholesterol, mevalonate pathway sterol intermediates, and their derivatives are critical downstream regulatory targets of lipid ferroptosis. The details of these findings are presented in Figure 3.

### 2.3 The Antioxidant Defense System: GPX4‐Centric and Non‐GPX4 Pathways

Acting as a counterbalance to the upstream iron‐driven lipid peroxidation cascade, the antioxidant defense system acts as the third core protective axis of ferroptosis. Functional collapse of this system is the key downstream event that transforms persistent lipid peroxidation into irreversible ferroptotic cell death. The ferroptosis‐related antioxidant network is a multilayered and redundant defense system composed of a dominant GPX4‐centric core pathway and multiple independent non‐GPX4 auxiliary pathways that together maintain cellular lipid redox homeostasis and limit the progression of ferroptosis.

The GPX4‐dependent metabolic pathway, formed by the Xc^—^–glutathione (GSH)–GPX4 axis, plays a key role in clearing lipid peroxides and is targeted by many molecules to modulate ferroptosis. System Xc^—^ dysfunction (via SLC7A11 or SLC3A2), intracellular GSH depletion, or GPX4 inactivation directly allow lipid peroxide accumulation and trigger ferroptosis [8, 49, 50]. Multiple transcription factors serve as key upstream regulatory factors that control the activity of this core axis by modulating SLC7A11 transcription. Under cell stress conditions, the transcription factor nuclear factor‐erythroid 2‐related factor 2 (NRF2) upregulates SLC7A11 expression to promote GSH biosynthesis and inhibit ferroptosis [51]. In contrast, p53 and ATF3 are negative regulatory factors: p53 transcriptionally downregulates SLC7A11 expression to facilitate ferroptosis [52], whereas ATF3 directly binds to the promotor of SLC7A11 to inhibit the expression of the gene and aggravate ferroptosis [53]. Accordingly, sustaining the structural and functional stability of the GSH–GPX4 core antioxidant system is essential for blocking ferroptosis execution.

To compensate for the functional deficiency of the GPX4 core pathway, three independent non‐GPX4 pathways—the FSP1/ubiquinol (CoQH_2_), dihydroorotate dehydrogenase (DHODH)/CoQH_2_, and GTP cyclohydrolase 1 (GCH1)/tetrahydrobiopterin (BH4) pathways—serve as auxiliary antioxidant nodes to form a complementary defense network. These three pathways inhibit ferroptosis independent of the Xc^—^–GSH–GPX4 axis, providing multilayered protection against lipid peroxidation. The FSP1/CoQH2 pathway is a major auxiliary antioxidant axis whose elements are distributed within the cytoplasmic membrane. Its core downstream protective effect relies on FSP1‐mediated redox reactions. Specifically, FSP1 utilizes NADPH as a cofactor to catalyze the reduction of CoQ10 to lipophilic CoQH2. CoQH_2_, as a lipophilic antioxidant, directly scavenges lipid radicals, effectively blocking the propagation of lipid peroxidation within the lipid bilayer and suppressing lipid peroxidation [54, 55]. Pharmacological inhibition (iFSP1) or activation (via compound 3f) affects the FSP1–CoQ10–NADPH pathway, potentially influencing sensitivity to ferroptosis [54, 56].

**FIGURE 4:**
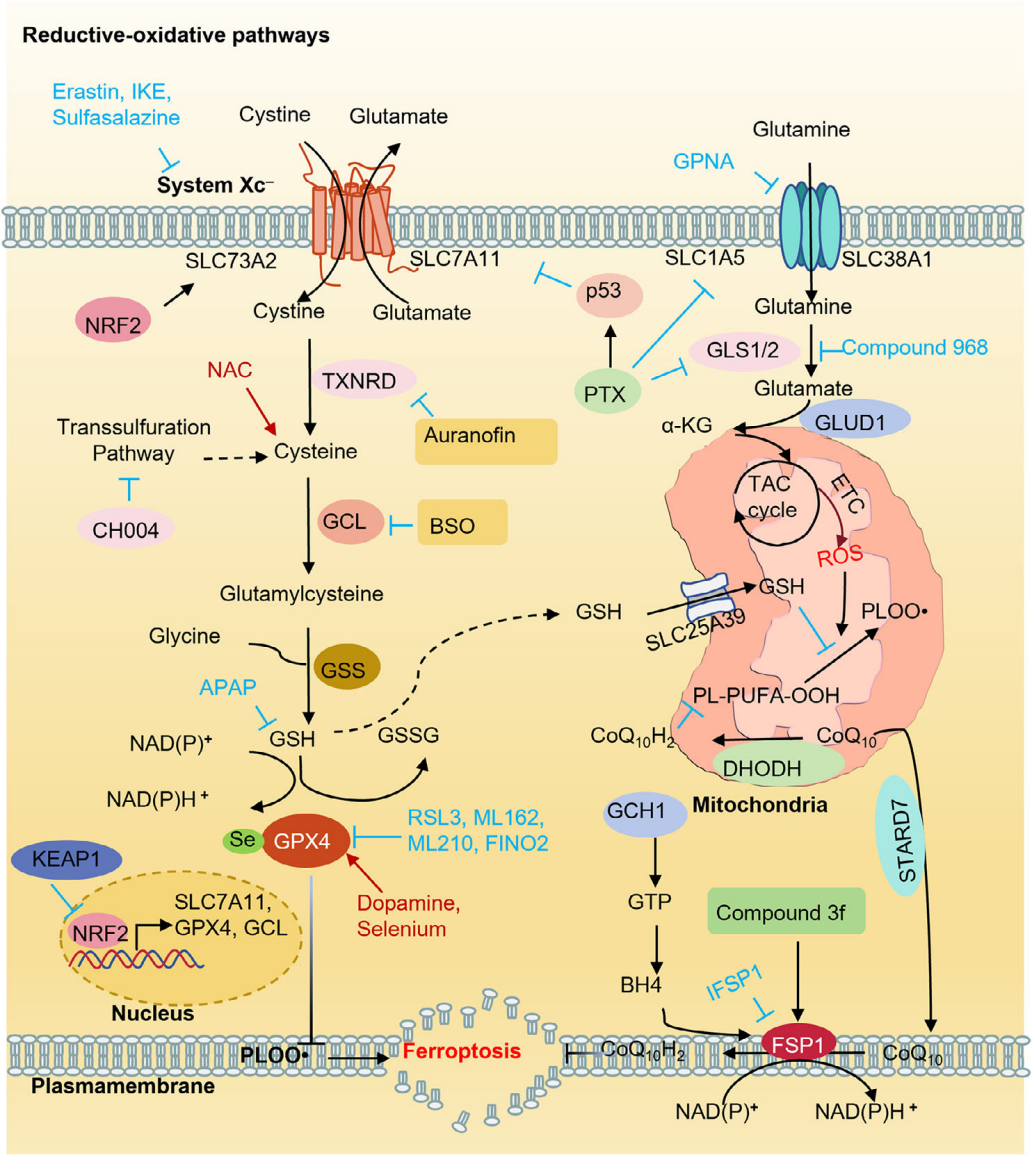
Targeting ferroptosis in reductive‐oxidative pathways. The ferroptosis‐related reductive‐oxidative pathways encompass the systemic Xc–GSH–GPX4 pathway, the FSP1–CoQ10–NADPH pathway, the mitochondrial DHODH‐mediated pathway, and the KEAP1–NRF2 antioxidant signaling pathway. SLC, solute carrier family; APAP, acetaminophen; NRF2, nuclear factor erythroid 2‐related factor 2; IKE, imidazole ketone erastin; NAC, N‐acetylcysteine; TXNRD, thioredoxin reductase; GCL, glutamate‐cysteine ligase; GSS, glutathione synthetase; GPX4, glutathione peroxidase 4; GSH, glutathione; GSSG, glutathione disulfide; DHODH, dihydroorotate dehydrogenase, DPP4 dipeptidyl peptidase 4; FSP1, ferroptosis suppressor protein 1; KEAP1, Kelch‐like ECH‐associated protein 1; TCA, tricarboxylic acid; GCH1, GTP cyclohydrolase 1; BH4, tetrahydrobiopterin; BSO, buthionine sulfoximine; PTX, paclitaxel; GPNA, L‐g‐glutamyl‐p‐nitroanilide; GLS, glutaminase; GLUD1, glutamate dehydrogenase 1; ETC, electrontransferchain; GTP, guanosine triphosphate; STARD7, StAR‐related lipid transfer protein 7; iFSP1, ferroptosis suppressor protein 1 inhibitor; SLC7A11, solute carrier family 7 member 11.

The mitochondrial DHODH/CoQH2 pathway acts as a mitochondria‐specific auxiliary antioxidant pathway. DHODH, a key enzyme in the pyrimidine synthesis pathway, also uses CoQ as an electron acceptor. During the oxidation of dihydroorotate to orotate, DHODH simultaneously reduces CoQ_10_ to CoQH_2_ [57]. This DHODH‐derived CoQH2 forms part of an independent mitochondrial antioxidant system that specifically eliminates mitochondrial lipid peroxides and compensates for the antioxidant deficiency that results from the inactivation of GPX4, thereby promoting resistance to mitochondrial ferroptosis. Another system that critically defends against ferroptosis is represented by the GCH1/BH4 pathway. GCH1, as the core rate‐limiting enzyme for BH4 synthesis, determines the activation of this pathway; BH4 and its oxidation products not only act as potent radical‐trapping antioxidants but also increase the antioxidant capacity of cellular membranes by promoting the synthesis of both CoQ_10_ and antioxidant MUFA phospholipids through multiple mechanisms [58]. However, the subcellular localization and compartment‐specific regulatory characteristics of the GCH1/BH4 system remain unclear and require further exploration. The details of the results described above are shown in Figure 4.

### 2.4 Ferroptosis and Other Forms of Cell Death

The iron accumulation‐mediated iron metabolic, the lipid peroxide accumulation‐driven lipid metabolic, and the glutathione/GPX4‐centered antioxidant pathways collectively define ferroptosis as a form of programmed cell death characterized by excessive iron‐dependent lipid peroxidation. This relatively novel form of programmed cell death is distinct from other forms of cell death, including apoptosis, autophagy, necroptosis, cuproptosis, and pyroptosis, in terms of morphological characteristics and core regulatory mechanisms. This paper summarizes the morphological features, inducers and inhibitors of ferroptosis, and other forms of cell death, as summarized in Table 1.

**TABLE 1:** Distinctions among cell death modes (1998–2025).

Types	Morphological changes	Positive	Negative	References
Apoptosis 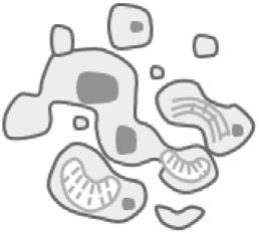	Cell shrinkage, nuclear condensation, nuclear membrane rupture, apoptotic body fragmentation, membrane integrity	Caspase family, BCL2 family (*BAX, BOK, BAK1, BBC3, BID, PMAIP1, BCL2L11*),	*BCL2, BCL2L1/2/10, MCL1*, IL‐4	[59, 60, 61]
Necroptosis 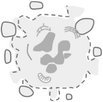	Cell swelling, chromatin condensation, membrane rupture, and leakage of contents	*RIPK1, RIPK3, MLKL, ZBP1*	cIAPs, ESCRT‐III, PPM1B, LUBAC, *AURKA*	[62, 63, 64]
Ferroptosis 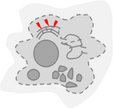	Mitochondrial condensation or swelling, increased membrane density, reduced cristae, and outer membrane rupture, lysosomal membrane rupture, plasma membrane rupture	*TFRC, ACSL4, GLS2, ALOX15, NCOA4, VDAC2/3, HSP90, PEBP1*	*GPX4, FSP1, NRF2, SLC7A11, FANCD2, HO‐1, SCD1, MUC1*	[65, 66, 67, 68, 69]
Pyroptosis 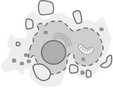	Cell swelling, membrane pore formation, plasma membrane rupture, release of proinflammatory cytokines	*Caspase1/11, GSDMD, NLRP3*, IL‐18, IL‐1β	PKA, ESCRTIII, *GPX4, NRF2*	[70, 71]
Autophagy 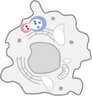	Cytoplasmic organelles enlargement or depletion, formation of intracellular autophagosomes, degradation of organelles, plasma membrane rupture	*BECN1, ATG5/7*, AMPK, *TFEB*	mTOR, *USP30*	[72, 73, 74, 75]
Cuproptosis 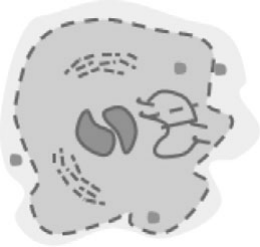	Mitochondrial contraction, cell membrane rupture, endoplasmic reticulum structure damage, chromatin fragmentation	*SLC31A1, DLAT, FDX1, LIPT1, PDHB, PDHA1, MELK*	*MTF1, GLS, CDKN2A, SLC7A11, ARID1A*	[4, 76, 77, 78, 79, 80]

**The main features, positive and negative of ferroptosis, apoptosis, autophagy, necroptosis, pyroptosis and cuproptosis**.

BCL2, B‐cell lymphoma 2; BAX, Bcl‐2‐associated X protein; BOK, BCL2‐related ovarian killer; BAK1, Bcl2 antagonist/killer 1; BBC3, BCL2 binding component 3; BID, BH3‐interacting domain death agonist; PMAIP1, phorbol‐12‐myristate‐13‐acetate‐induced protein 1; MCL1, myeloid cell leukemia 1; IL‐4, interleukin‐4; RIPK1/3, receptor interacting serine/threonine kinase 1/3; MLKL, mixed lineage kinase domain‐like pseudokinase; ZBP1, Z‐DNA binding protein 1; ESCRT‐III, endosomal sorting complex required for transport III; PPM1B, protein phosphatase 1B; LUBAC, linear ubiquitin chain assembly complex; AURKA, aurora kinase A; TFRC, transferrin receptor; ACSL4, acyl‐CoA synthetase long chain family member 4; GLS2, glutaminase 2; ALOX15, arachidonate‐15‐lipoxygenase; NCOA4, nuclear receptor coactivator 4; VDAC2/3, voltage‐dependent anion channel 2/3; HSP90, heat shock protein 90; PEBP1, phosphatidylethanolamine binding protein 1; GPX4, glutathione peroxidase 4; FSP1, ferroptosis suppressor protein 1; NRF2, nuclear factor erythroid 2‐related factor 2; SLC7A11, solute carrier family 7 member 11; FANCD2, Fanconi anemia complementation group D2; HO‐1, hemeoxygenase‐1; SCD1, stearoyl‐CoA desaturase1; MUC1, mucin 1; GSDMD, gasdermin D; NLRP3, NLR family, pyrin domain containing protein 3; PKA, protein kinase A; ESCRTIII, endosomal sorting complex required for transport III; BECN1, beclin 1; ATG5/7, autophagy‐related 5/7; AMPK, adenosine 5‘‐monophosphate (AMP)‐activated protein kinase; TFEB, transcription factor EB; mTOR, mammalian target of rapamycin; USP30, ubiquitin‐specific protease 30; SLC31A1, the solute carrier family 31 member 1; DLAT, dihydrolipoamide S‐acetyltransferase; FDX1, ferredoxin 1; LIPT1, lipoyl transferase 1; PDHB, pyruvate dehydrogenase E1 subunit beta; PDHA1, pyruvate dehydrogenase E1 subunit alpha 1; MELK, maternal embryonic leucine zipper kinase; MTF1, metal‐regulatory transcription factor‐1; GLS, glutaminase; CDKN2A, cyclin‐dependent kinase inhibitor 2A; SLC7A11, solute carrier family 7 membrane 11; ARID1A, AT‐rich interactive domain 1A.

In vivo, the different forms of cell death do not act independently but rather exhibit distinct mechanistic differences and intricate crosstalk, thereby coordinately modulating cell fate, tissue homeostasis, and disease initiation and progression. Ferroptosis cooperates with other forms of cell death through complex regulatory networks [81, 82, 83, 84, 85]. For example, ferroptosis can promote cellular sensitivity to apoptosis. The tumor suppressor gene p53 can eliminate tumor cells through cell cycle arrest and apoptosis, and can induce ferroptosis in tumor cells [86]. ACSL4 deficiency, a marker of ferroptosis, leads to increased expression of MLKL, a marker of necroptosis, indicating the complementarity of ferroptosis and necroptosis [87, 88]. Additionally, studies have shown that the occurrence of ferroptosis depends on the occurrence of autophagy, and many ferroptosis regulators have been identified as potential autophagy factors [89]. NCOA4‐mediated ferritinophagy can promote iron release and increase the concentration of free iron in cells, whereas NCOA4 knockdown or knockout of NCOA4 can inhibit ferroptosis [90]. Collectively, the results of these studies show that ferroptosis not only has distinct morphological and biochemical hallmarks but also is incorporated into a broad regulatory landscape involving apoptosis, necroptosis, and autophagy. These interwoven cell death pathways form a sophisticated network that governs cell fate decisions in both physiological and pathological contexts.

## 3 Ferroptosis as a Pathogenic Driver in Human Diseases

Ferroptosis is a unique, highly regulated form of iron‐dependent cell death. Physiologically, ferroptosis is involved in sustaining immune homeostasis, remodeling tissues, and suppressing tumors to maintain organismal health. However, aberrant ferroptosis disturbs cellular redox homeostasis, triggers inflammatory cascades, and causes irreversible tissue damage and organ dysfunction. Accumulating evidence has indicated that ferroptosis dysregulation serves as a crucial driver of numerous human diseases, including cancer, NDDs, cardiovascular disorders, chronic inflammation, and acute organ injury. Elucidating the mechanistic association of ferroptosis with disease progression could improve our understanding of disease development and yield novel therapeutic targets. This chapter elaborates on the molecular mechanisms that underlie ferroptosis‐driven pathological changes across multiple diseases, summarizes recent research progress, and highlights the potential of ferroptosis‐targeted strategies for translation into clinical diagnostics and precision therapeutics.

### 3.1 Cancer: The Double‐Edged Sword of Ferroptosis

**FIGURE 5:**
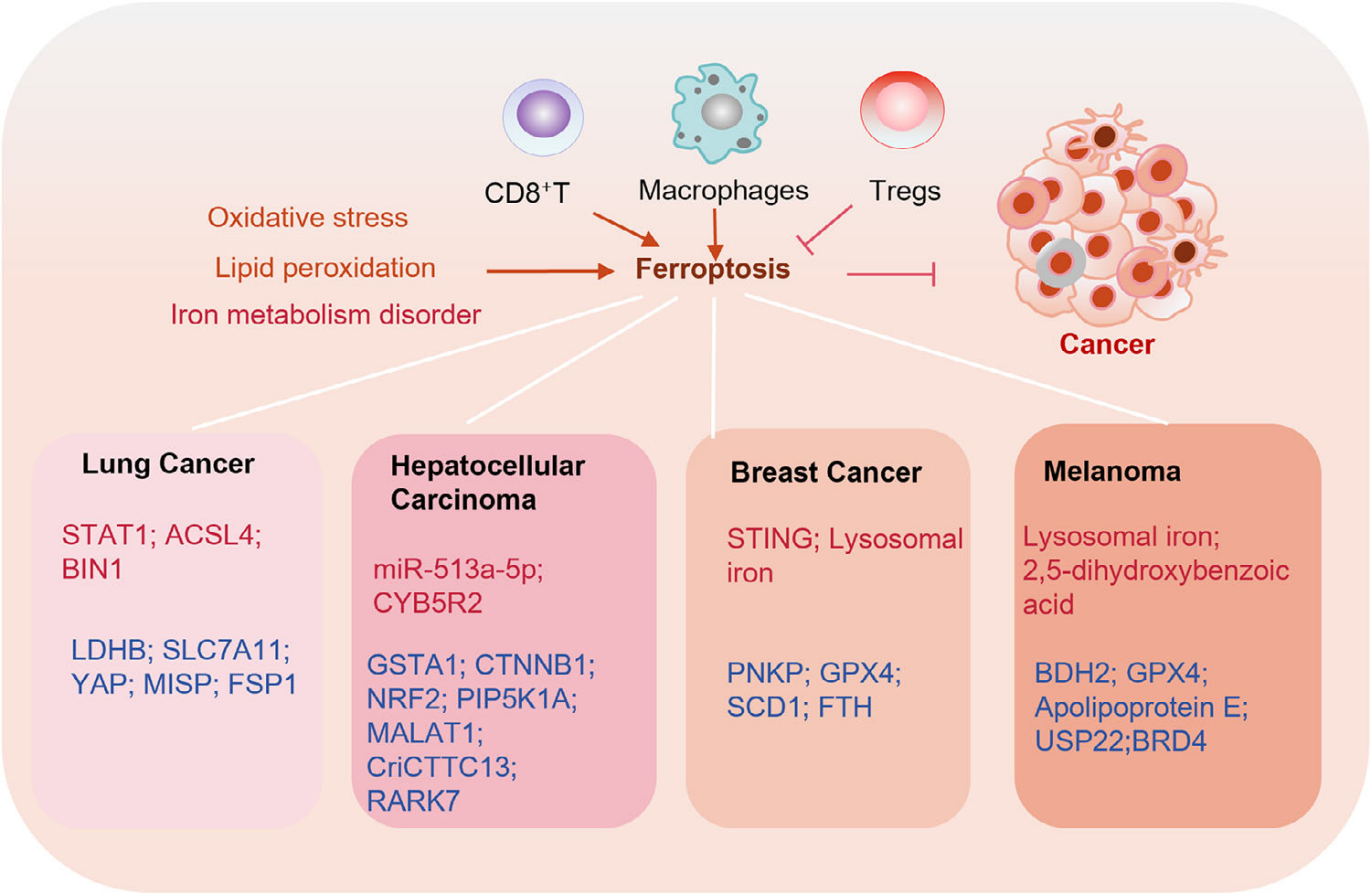
Ferroptosis regulation and tumor development. The perspective of ferroptosis in lung cancer, hepatocellular carcinoma, breast cancer, and melanoma. STAT1, transducer and activator of transcription 1; ACSL4, acyl‐CoA synthetase‐4; SLC7A11, solute carrier family 7 member 11; LDHB, recombinant lactate dehydrogenase B; YAP, Yes‐associated protein; MISP, mitotic spindle–positioning protein; FSP1, ferroptosis suppressor protein 1; CYB5R2, cytochrome b5 reductase 2; GSTA1, glutathione S‐transferase alpha 1; CTNNB1, catenin (cadherin‐associated protein), beta 1; PIP5K1A, phosphatidylinositol 4‐phosphate 5‐kinase 1 alpha; MALAT1, metastasis‐associated lung adenocarcinoma transcript 1; RARK7, Parkinsonism‐associated deglycase 7; STING, stimulator of interferon genes; PNKP, polynucleotide kinase/phosphatase; SCD1, stearoyl‐CoA desaturase‐1; GPX4, glutathione peroxidase 4; FTH, ferritin heavy chain; BDH2, 3‐hydroxybutyrate dehydrogenase 2; USP22, ubiquitin‐specific protease 22; BRD4, bromodomain‐containing protein 4.

Ferroptosis has been intrinsically linked to cancer biology since its original characterization, owing to the identification of its chemical inducers in the identification of anticancer compounds [6, 91]. In recent years, accumulating evidence has indicated that ferroptosis is a critical regulatory mechanism in the pathogenesis and progression of multiple malignancies, including lung cancer [92], hepatocellular carcinoma (HCC) [93], breast cancer [94], and melanoma [95]. This review comprehensively synthesizes the current understanding of the molecular mechanisms by which ferroptosis is involved in the progression of several types of cancer and further explores its potential applications as a therapeutic target in cancer immunotherapy; a summary of the results is provided in Figure 5.

#### 3.1.1 Lung Cancer

Lung cancer is the most commonly diagnosed malignancy and the leading cause of cancer‐related mortality worldwide, with approximately 2.48 million new cases and 1.8 million deaths reported globally in 2022 [96, 97]. Despite substantial progress in its diagnosis and treatment, advanced lung cancer remains associated with unsatisfactory patient outcomes, underscoring the need to further understand its molecular basis and identify novel therapeutic targets [98, 99]. In lung cancer, ferroptosis susceptibility is predominantly governed by two conserved antioxidant regulatory axes: the canonical SLC7A11–GPX4 pathway and the alternative FSP1–CoQ10 pathway. Most reported regulatory molecules exert pro‐ or antiferroptotic functions via modulating the activity of these two core signaling cascades. In KRAS‐mutant lung cancer, lactate dehydrogenase B (LDHB) functions as a suppressor of ferroptosis by inhibiting the activation of signal transducer and activator of transcription 1 (STAT1) and sustaining the SLC7A11–GSH antioxidant axis. LDHB knockdown promotes glutaminolysis and mitochondrial oxidative stress, ultimately inducing systemic lethal ferroptosis [100]. Moreover, ferroptotic stress‐induced SUMO2–K11 lactylation promotes ACSL4 degradation, thereby limiting lipid peroxidation and increasing ferroptosis resistance in lung adenocarcinoma cells [101]. In non‐small cell lung cancer, mitochondrial spindle‐positioning protein potentiates the activity of the SLC7A11–GSH antioxidant axis by activating YAP‐dependent SLC7A11 transcription, whereas bridging integrator 1 deficiency attenuates ferroptotic responses through the suppression of STAT1 signaling and GSH metabolism, thereby promoting tumor growth and immune evasion [102, 103]. Alongside the GPX4‐dependent antioxidant system, the ferroptosis suppressor protein 1 (FSP1)–coenzyme Q_10_ (CoQ_10_) axis represents a major parallel ferroptosis‐defense pathway in lung cancer. By reducing CoQ10 to its radical‐trapping form, FSP1 prevents lipid peroxidation and preserves redox homeostasis. Consequently, FSP1 deficiency sensitizes lung cancer cells to ferroptosis and suppresses tumor growth, whereas high FSP1 expression is associated with poor patient outcomes. Accordingly, selective FSP1 inhibitors have emerged as promising therapeutic agents in preclinical lung cancer models [104]. Collectively, these findings indicate that ferroptosis represents a critical, potential therapeutic target in lung cancer, particularly in KRAS‐mutant tumors, which exhibit profound metabolic reprogramming and elevated oxidative stress. Key regulatory pathways, including the SLC7A11–GSH–GPX4 axis and the FSP1–CoQ10 defense system, allow tumor cells to evade ferroptotic cell death and sustain disease progression. Targeting these ferroptosis‐protective mechanisms may increase the efficacy of conventional therapies, overcome drug resistance, and improve antitumor immune responses. Therefore, ferroptosis‐based therapeutic strategies hold considerable promise for treating lung cancer, especially for patients with KRAS‐mutant and treatment‐refractory disease.

#### 3.1.2 Hepatocellular Carcinoma

HCC is a leading cause of cancer‐related mortality worldwide. Accumulating evidence suggests that sorafenib resistance is not driven by a single molecular alteration but results from the coordinated suppression of ferroptosis through metabolic reprogramming, improved antioxidant defenses, and epigenetic regulation. Therefore, restoring ferroptosis sensitivity has emerged as a promising strategy for overcoming this resistance and improving therapeutic outcomes in patients with HCC [96, 105, 106, 107]. At the metabolic level, the upregulation of glutathione s‐transferase alpha 1 (GSTA1) markedly increases peroxidative detoxification, thereby reducing lipid peroxidation at its source and attenuating sorafenib‐induced ferroptotic stress. This metabolic safeguard is sustained through the catenin beta 1 (CTNNB1)‐dependent transcriptional activation of GSTA1 and a positive feedback loop that promotes CTNNB1 nuclear translocation [108]. Simultaneously, phosphatidylinositol‐4‐phosphate 5‐kinase type 1 alpha (PIP5K1A) stabilizes NRF2 by competitively binding to kelch‐like ECH‐associated protein 1 (KEAP1), allowing persistent antioxidant gene program activation and further suppressing the propagation of lipid peroxidation; pharmacologic inhibition of PIP5K1A disrupts this network and restores cellular susceptibility to ferroptosis [109]. Concurrently, RNA stability and epigenetic regulation form an additional layer of ferroptosis suppression. NOP2/Sun RNA methyltransferase 2 (NSUN2)‐mediated m5C methylation and Aly/REF export factor (ALYREF)‐dependent RNA export maintain the high expression of metastasis‐associated lung adenocarcinoma transcript 1 (MALAT1), while MALAT1 facilitates cytoplasmic translocation of ELAV‐like RNA binding protein 1 (ELAVL1), which stabilizes the mRNA of the key ferroptosis suppressor SLC7A11, thereby sustaining cystine uptake and glutathione homeostasis [110]. Similarly, circTTC13 increases SLC7A11 expression by sequestering miR‐513a‐5p, allowing cancer cells to maintain redox balance under iron‐dependent oxidative stress conditions and acquire more aggressive and drug‐resistant phenotypes [111]. Additionally, the activating transcription factor 7‐interacting protein (ATF7IP)‐SET domain bifurcated histone lysine methyltransferase 1 (SETDB1) complex represses cytochrome B5 reductase 2 (CYB5R2) and stabilizes the antioxidant protein Parkinsonism‐associated deglycase (PARK7), lowering intracellular Fe^2+^ concentrations and promoting glutathione synthesis and ultimately decreasing ferroptotic vulnerability [112]. Taken together, the results of these studies support a complex mechanistic principle: sorafenib resistance arises through the coordinated engagement of multiple regulatory pathways that collectively suppress ferroptosis. Thus, therapeutic strategies designed to dismantle this integrated resistance network and reinstate ferroptosis sensitivity may offer a translationally meaningful method for improving treatment responses and clinical outcomes in patients with HCC.

#### 3.1.3 Breast Cancer

Breast cancer is among the most common and life‐threatening malignant tumors among women worldwide. According to GLOBOCAN 2022, more than 2.3 million new cases of breast cancer are diagnosed globally each year, accounting for 11.6% of all cancer cases, and more than 660,000 deaths occur annually, making breast cancer the fourth leading cause of cancer‐related mortality [96, 113, 114]. In recent years, with advances in the understanding of tumor biology, increasing attention has been given to various methods of RCD beyond apoptosis. Among them, ferroptosis has emerged as a distinct iron‐dependent form of cell death driven by lipid peroxidation and iron metabolism imbalance. The growing understanding of its relevance in the initiation, progression, and treatment of breast cancer has provided new insights and potential strategies for therapeutic intervention in the disease [115]. Recent studies have redefined the contribution of lysosomes to ferroptosis, identifying lysosomal iron as a pivotal trigger of this form of cell death. The acidic lysosomal milieu facilitates iron‐dependent Fenton chemistry, initiating phospholipid peroxidation within the membranes of lysosomes and driving oxidative damage that subsequently propagates throughout the cell. These insights have further revealed that liproxstatin‐1 primarily acts by chelating lysosomal iron rather than solely by scavenging lipid radicals. In contrast, the bifunctional agent fentomycin‐1 can accumulate within lysosomes and potentiate iron reactivity, thereby inducing ferroptosis with pronounced selectivity toward iron‐loaded, CD44‐high drug‐resistant and metastatic tumor cells. This lysosome‐centered strategy has demonstrated substantial antitumor efficacy across multiple models of breast cancer and functions synergistically with standard chemotherapies, underscoring its potential as a therapeutic approach for otherwise refractory malignancies [116]. Moreover, studies on regulating ferroptosis have identified the polynucleotide kinase 3′‐phosphatase (PNKP)‐STING (stimulator of interferon genes) axis as another critical pathway in triple‐negative breast cancer (TNBC). PNKP is highly expressed in TNBC, where it is associated with poor patient outcomes and resistance to doxorubicin (DOX)‐resistant melanocyte‐inducing transcription factor (MITF); loss of PKNP markedly increases intracellular iron loading and ROS levels while reducing the expression of GPX4, SCD1, and FTH, thereby increasing lipid peroxidation and tumor susceptibility to ferroptosis. Further investigations have revealed that the downregulation of PNKP activates STING signaling and promotes transcription factor EB (TFEB)‐driven lysosomal biogenesis and ferritinophagy, whereas the dephosphorylation of STAT3 potentiates this autophagy–lysosome–ferroptosis regulatory network. Ultimately, inhibition of PNKP combined with DOX administration achieves a pronounced synergistic effect in cell lines, patient‐derived organoids, and animal models, resulting in tumor suppression through a dual mechanism involving DNA damage and ferroptosis and thus offering a promising therapeutic strategy for drug‐resistant TNBC [117]. Taken together, these findings suggest that lysosomes, by virtue of their unique iron‐rich and acidic microenvironments, act as key initiators of ferroptosis and as strategic targets of therapies that combine the regulation of iron metabolism, autophagy, and lipid peroxidation in anticancer interventions.

#### 3.1.4 Melanoma

Melanoma is a highly aggressive malignancy that originates from melanocytes and is characterized by marked genetic heterogeneity and cellular plasticity, features which collectively contribute to high metastatic potential and complex clinical behavior [118]. Although notable treatment progress has been made through the development of targeted therapies and immune checkpoint inhibitors, the global incidence of melanoma continues to increase, and therapeutic resistance remains widespread, with approximately 70–90% of patients with advanced disease eventually experiencing relapse. In addition, the toxicity associated with combination immunotherapy also limits the durability of its clinical benefits [119]. Therefore, a deeper understanding of the underlying molecular mechanisms and the basis of treatment resistance in melanoma is highly important.

Ferroptosis is an iron‐dependent form of RCD driven by lipid peroxidation. It plays a dual role in melanoma progression, invasion, and therapeutic resistance, acting both as an intrinsic vulnerability in tumor cells while influencing treatment efficacy through its modulation by the tumor microenvironment [120]. Recent studies have shown that the sensitivity to ferroptosis is governed by multilayer regulatory networks that involve cellular state plasticity, intercellular communication, and epigenetic regulation. As melanoma cells transition from a MITF (high) proliferative identity to an MITF (low) mesenchymal state, they undergo profound subcellular iron partitioning reprogramming: BDH2 knockout disrupts the 2,5‐dihydroxybenzoic acid‐dependent iron shuttle at sites of mitochondria‒lysosome contact, leading to lysosomal iron sequestration, impaired mitochondrial respiration, and greater susceptibility to ferroptosis [121]. This intrinsic vulnerability is further regulated by paracrine signaling within the heterogeneous tumor microenvironment. Proliferative MITF (high) melanoma cells secrete apolipoprotein E via a USP22‐associated pathway, resulting in remodeling of the PUFA‐enriched phospholipid composition and the upregulation of GPX4 activity in neighboring invasive cells, ultimately establishing a cross‐cell state ferroptosis‐resistance network [122]. Moreover, melanoma cells further enhance ferroptosis evasion through multiple molecular mechanisms, including the SIRT1–PTEN–PI3K signaling axis, which promotes lipid desaturation and increases antioxidant capacity, and the bromodomain‐containing 4 (BRD4)–AKR1C2 epigenetic regulatory axis, which facilitates the detoxification of lipid peroxides [123, 124]. Notably, inhibition of USP22 or BRD4 expression can restore ferroptosis sensitivity in tumor cells and increase the efficacy of both GPX4‐targeted therapies and immune checkpoint blockade. Overall, these findings suggest that ferroptosis can be characterized by a highly dynamic, cell state‐dependent vulnerability shaped by lineage transitions, tumor microenvironmental signaling, and chromatin regulatory networks, thereby providing a theoretical basis for multitarget combination therapeutic strategies.

From a pan‐cancer perspective, ferroptosis regulation is relatively well conserved across lung cancer, HCC, breast cancer, and melanoma. Tumor cells typically rely on increased SLC7A11‐mediated cystine uptake and GSH synthesis and coordination among antioxidant systems such as those governed by GPX4 and FSP1 to suppress lipid peroxidation, thereby evading ferroptosis and ensuring survival. This core antiferroptosis network represents a shared adaptive survival mechanism across multiple tumor types. Similarly, iron metabolism reprogramming, redox homeostasis regulation, and noncoding RNA as well as epigenetic mechanisms exhibit some degree of conservation across cancers, suggesting a relatively unified framework underlying ferroptosis escape in these diseases. All this said, tumor type‐specific differences in ferroptosis regulation are evident, largely driven by tissue‐of‐origin metabolic features and unique tumor microenvironmental contexts. Accordingly, the ferroptosis regulatory landscape can be summarized as a hierarchical architecture comprising a conserved, core module with antioxidant characteristics yet diverse upstream regulatory layers. This combination of shared and context‐dependent features not only helps explain differences in ferroptosis sensitivity among tumor types but also provides a theoretical foundation for the development of stratified, precise ferroptosis‐targeted therapeutic strategies.

#### 3.1.5 Ferroptosis in the Tumor Immune Microenvironment

Beyond the tissue‐specific ferroptotic features observed in individual cancer subtypes, ferroptosis‐mediated remodeling of the tumor immune microenvironment (TME) acts as a universal regulatory mechanism shared across most malignancies. This crosstumor conserved pathway underlies the dual immunostimulatory and immunosuppressive outcomes of ferroptosis in tumor progression [125]. When they undergo ferroptosis, tumor cells release a series of damage‐associated molecular patterns (DAMPs), including HMGB1, ATP, and nucleic acids, as well as multiple lipid metabolites, such as oxidized phospholipids, PGE2, and 4‐HNE, which trigger both innate and adaptive immune responses [126, 127]. However, this ferroptosis‐triggered immune activation has complex, dual effects on the tumor microenvironment. First, ferroptosis can deplete immunosuppressive cells such as regulatory T cells (Tregs), myeloid‐derived suppressor cells, and M2‐polarized tumor‐associated macrophages (TAMs), thus alleviating immune suppression and potentiating antitumor responses [128]. Second, the survival and function of critical immunostimulatory cells—including CD8^+^ T cells and dendritic cells—are dependent on robust ferroptosis defense systems (GPX4 and SLC7A11); their impairment via excessive ferroptosis leads to immune dysfunction and evasion. This dual functionality underscores the context‐dependent nature of ferroptosis in cancer progression and treatment responses.

The TME can be characterized by a highly dynamic interplay between immune cell function and ferroptosis, wherein distinct immune cell populations harbor distinct vulnerabilities and resistance mechanisms [129]. CD8^+^ T cells, particularly those infiltrating tumors, are markedly susceptible to ferroptosis because of their increased metabolic activity and lipid peroxidation levels [130]. This vulnerability is exacerbated by the presence of immunosuppressive factors such as PD‐1 signaling and the upregulation of the expression of the lipid uptake receptor CD36, which jointly increase the concentrations of intracellular ROS and promote lipid peroxidation. In mouse models, ACSL4 knockout protected CD8^+^ T cells from ferroptosis but simultaneously impaired their antitumor efficacy, indicating the presence of a delicate balance between survival and functional competence. Similarly, exhausted CD8^+^ T cells (TEXs) undergoing chronic antigen stimulation exhibit increased lipid peroxidation and mitochondrial oxidative stress, making them more susceptible to ferroptosis [131, 132]. Conversely, Tregs may protect tumor cells from ferroptosis by secreting IL‐10 and TGF‐β or generating metabolic precursors such as glutathione [133]. Furthermore, ferroptosis‐associated signaling molecules such as HMGB1 and 8‐OHG promote the polarization of TAMs toward the proinflammatory M1 phenotype; in contrast, KRASG12D tends to induce the formation of M2‐type macrophages [134]. Notably, compared with M2‐type macrophages, M1‐type macrophages are more resistant to ferroptosis, a phenomenon attributed to the high expression of inducible nitric oxide synthase in M1 cells, which can replace GPX4 as a negative regulator of ferroptosis. These findings collectively reveal a dynamic “immune–ferroptosis axis,” whose bidirectional regulatory mechanisms profoundly influence tumor progression and therapeutic response.

Diverse therapeutic strategies that target ferroptosis–immune crosstalk, including nanocarrier delivery systems, natural bioactive compounds, and targeted genetic intervention, are currently available [135]. Nanoplatforms enable precise and localized delivery of ferroptosis inducers, whereas microneedle‐based transdermal drug delivery suppresses aggressive TNBC progression via two mechanisms: Cu^2+^ chelation alleviates TME immunosuppression, while metal ion‐triggered Fenton reactions potentiate ferroptosis [136]. Mannosylated nanoparticles selectively target TAMs, induce TAM‐based ferroptosis, reverse M2‐type immunosuppressive polarization, and improve the efficacy of anti‐PD‐L1 therapy [137]. Natural products such as curcumin, gallic acid, and epigallocatechin gallate (EGCG) modulate ferroptosis by regulating autophagy, tsRNA‐13502, and the Nrf2/SLC7A11/GPX4 axis, exhibiting multitarget, antitumor potential [138, 139]. SLC7A11 is a core regulator of ferroptosis; its overexpression drives tumor resistance to ferroptosis. Interventions targeting the inhibition of SLC7A11 or L‐selenocysteine effectively induce oxidative stress and ferroptotic cell death in tumor cells [138, 139]. Furthermore, cancer cells with a high mesenchymal phenotype are highly sensitive to ferroptosis and can be eliminated through a combination of statins and ferroptosis inducers can efficiently eliminate this drug‐resistant cell subpopulation [140]. In summary, ferroptosis engages in complex bidirectional crosstalk with the TME, resulting in both immunostimulatory positive feedback effects and paradoxically immunosuppression. Multiple combination therapies based on ferroptosis can effectively remodel the TME, overcome therapeutic resistance in tumors, and improve systemic antitumor immunity, providing a solid theoretical basis and broad clinical translational prospects for precision tumor immunotherapy.

### 3.2 Cardiovascular Diseases

**FIGURE 6:**
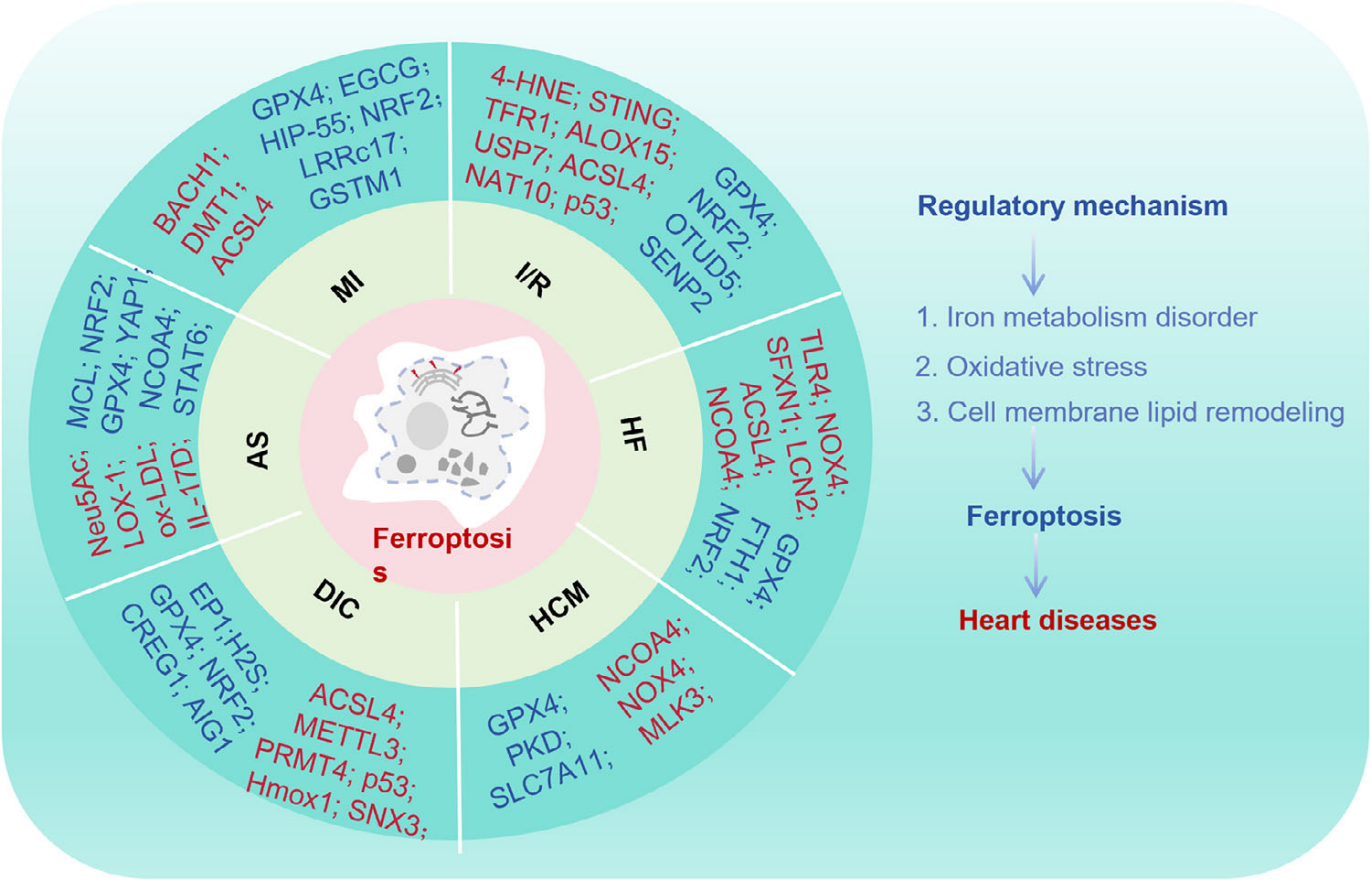
Ferroptosis in cardiovascular disease. Ferroptosis is closely related to the development of cardiovascular diseases such as myocardial infarction (MI), myocardial ischemia–reperfusion injury (I/R), doxorubicin‐induced cardiotoxicity (DIC), atherosclerosis (AS), hypertrophic cardiomyopathy (HCM), and heart failure (HF). GPX4, glutathione peroxidase 4; EGCG, epigallocatechin gallate; HIP‐55, hematopoietic progenitor kinase‐interacting protein of 55 kDa; NRF2, nuclear factor erythroid 2‐related factor 2; LRRc17, leucine‐rich repeat protein 17; GSTM1, glutathione S‐transferase M1; BACH1, BTB and CNC homolog 1; DMT1, divalent metal transporter 1; ACSL4, acyl‐CoA synthetase‐4; 4‐HNE, 4‐hydroxynonenal; STING, stimulator of interferon genes; TFR1, transferrin receptor 1; ALOX15, arachidonate 15‐lipoxygenase‐1; USP7, ubiquitin‐specific protease‐7; NAT10, N‐acetyltransferase 10; OTUD5, ovarian tumor deubiquitinase 5; SENP2, SUMO‐specific peptidase 2; TLR4, Toll‐like receptor 4; NOX4, NADPH oxidase 4; SFXN1, sideroflexin1; LCN2, lipocalin‐2; NCOA4, nuclear receptor coactivator 4; FTH1, ferritin heavy chain 1; MLK3, mixed lineage kinase 3; PKD, protein kinase D; SLC7A11, solute carrier Family 7 member 11; METTL3, methyltransferase‐like 3; PRMT4, protein arginine methyltransferase 4; SNX3, sorting nexin 3; EP1, E‐prostaglandin 1 receptor; H2S, hydrogen sulfide; CREG1, cellular repressor of E1A‐stimulated genes; AIG1, androgen‐induced gene 1; Neu5Ac, N‐acetylneuraminic acid; OX‐LDL, oxidized low‐density lipoprotein; LOX, lipoxygenase; IL17D, recombinant Interleukin 17D; MCL, micheliolide; YAP1, yes‐associated protein 1; STAT6, signal transducer and activator of transcription 6.

Cardiovascular disease is a prevalent disorder that poses a substantial threat to human health, whose incidence increases with age. Despite notable progress in basic research on this group of conditions, the pathogenic mechanisms that underly cardiovascular diseases remain incompletely understood. Cardiomyocytes are terminally differentiated cells with limited proliferative and regenerative capacity; consequently, cardiomyocyte loss results in a reduced viable cell pool, leading to structural remodeling and functional impairment of the heart and ultimately contributing to heart failure (HF) onset and progression [141, 142, 143]. Therefore, inhibiting cardiomyocyte death is a critical but challenging task in current cardiovascular disease research. Accumulating evidence suggests that ferroptosis is closely related to the development of cardiovascular diseases such as myocardial infarction (MI), myocardial ischemia–reperfusion (I/R) injury, drug‐induced cardiotoxicity, diabetic cardiomyopathy, and HF [144, 145], but the specific mechanisms that underlie their pathogenic processes are not fully understood. Therefore, here, we summarize the mechanisms by which ferroptosis regulates cardiovascular diseases that have been elucidated in recent years to provide new bases for research on cardiovascular diseases; details of these results are presented in Figure 6.

#### 3.2.1 Ferroptosis and MI

MI is a disease characterized by irreversible cardiomyocyte death caused by persistent local myocardial ischemia and hypoxia following coronary artery occlusion. In this context, ferroptosis acts as a core pathological mechanism that mediates myocardial injury post‐MI. Three interconnected mechanisms form the core of MI‐induced ferroptosis: iron overload (exacerbated by DMT1‐mediated ferrous iron influx), GPX4 downregulation (the key initiator of antiferroptosis activity), and NRF2 signaling impairment (which reduces the transcription of xCT, GPX4, and Hmox1).

GPX4 serves as the core effector of cardiomyocyte ferroptosis. In MI models, significant downregulation of GPX4 expression induces cysteine depletion, metabolic disturbance, and subsequent cardiomyocyte ferroptosis, thereby exacerbating myocardial damage [145]. Administration of the iron chelator DFO reduces iron accumulation in infarcted tissues and suppresses ferroptosis; this iron metabolism‐targeted strategy has been verified preclinically and has considerable translational value for managing MI [146]. The NRF2 signaling pathway functions as the central upstream regulator of GPX4. *Salvia miltiorrhiza* activates this pathway, upregulates the expression of xCT and GPX4, decreases ferrous ion and MDA levels, and ultimately inhibits post‐MI ferroptosis to alleviate myocardial injury [147]. Similarly, activation of the NRF2/heme oxygenase 1 (Hmox1) pathway suppresses MI‐triggered ferroptosis and exerts cardioprotective effects [148]. In contrast, BTB and CNC homolog 1 (BACH1), key regulators of iron metabolism, suppress the transcription of antiferroptotic genes, specifically exacerbating cardiomyocyte ferroptosis and acute MI deterioration, and thus act as critical negative regulators in ischemic myocardium [149]. Independent of the canonical NRF2/GPX4 axis, multiple bypass signaling pathways and exogenous regulators modulate MI‐related ferroptosis and have shown favorable translational potential. Human umbilical cord mesenchymal stem cell‐derived exosomes inhibit divalent metal‐ion transporter‐1 (DMT1)‐mediated ferrous ion influx, block cardiomyocyte ferroptosis, and mitigate MI injury, indicating potential as a cell‐free therapeutic approach [150].

Cinnamaldehyde and EGCG are natural, active compounds that suppress myocardial lipid peroxidation and ferroptosis by activating the PI3K/AKT pathway and modulating the miR‐450b‐5p/ACSL4 pathway, respectively, providing solid preclinical evidence for the development of natural drugs against MI [151, 152]. Moreover, the adaptor protein hematopoietic progenitor kinase 1 (HPK1)‐interacting protein of 55 kDa (HIP‐55) activates the mitogen‐activated protein kinase kinase kinase kinase 1 (MAP4K1)/c‐Jun N‐terminal kinase (JNK) cascade to upregulate the expression of GPX4, forming a noncanonical bypass antiferroptotic mechanism that can protect against MI damage [153]. Ferroptosis also contributes to long‐term post‐MI complications by mediating myocardial fibrosis and ventricular remodeling, which profoundly affect patient outcomes. Leucine‐rich repeat protein 17 (LRRc17) affects ventricular remodeling after MI by mediating myocardial ferroptosis and regulating myocardial fibrosis after MI [154], thus acting as a vital link between ferroptosis and pathological cardiac remodeling. The results of multiomic analyses have revealed that glutathione S‐transferase M1 (GSTM1) inhibits myocardial fibrosis after MI by inhibiting lipid peroxidation and ferroptosis [155], providing a novel translational target for precise interventions against postinfarction fibrosis. In summary, ferroptosis occurs throughout the entire pathological process of acute myocardial injury and post‐MI chronic fibrosis. The ferroptosis regulatory network centered on the NRF2/GPX4 axis and coordinated by multiple bypass pathways critically drives MI progression. Accumulating preclinical evidence has indicated that ferroptosis‐related targets and intervention strategies hold great translational potential for the development of novel anti‐MI therapeutic agents.

#### 3.2.2 Ferroptosis and Myocardial I/R Injury

I/R injury is a serious clinical complication that can affect organs such as the heart, liver, and brain [156, 157]. I/R‐induced ferroptosis is driven primarily by iron overload and lipid peroxidation, which both lead to the inactivation or degradation of GPX4, the core effector of this form of cell death, while dysregulation of upstream signaling pathways (e.g., the NRF2/Hmox1, P53, and cGAS–STING pathways) and posttranslational modifications further exacerbate this process.

Iron overload and excessive lipid ROS accumulation are two key triggers of I/R‐related ferroptosis [158, 159]. The results of clinical studies involving cardiovascular magnetic resonance imaging have shown that myocardial iron around the area of acute infarction is a major risk factor for adverse left ventricular remodeling in patients, demonstrating the clinical relevance of iron dysregulation in the pathogenesis of I/R injury [160]. TfR1, a key iron homeostasis regulator, mediates iron overload to initiate ferroptosis. In a rat I/R injury model, ubiquitin‐specific protease‐7 (USP7) upregulation activated P53 and further increased TfR1 expression, thereby promoting cardiomyocyte ferroptosis and I/R injury [161]. Targeting iron overload and TfR1‐mediated iron influx has reliable protective effects in preclinical trials and thus good translational potential as a form of I/R intervention.

GPX4‐mediated lipid peroxide clearance is the core effector mechanism that mitigates I/R injury ferroptosis. Mitochondrial GPX4 overexpression effectively eliminates lipid ROS and confers cardioprotection against I/R injury, confirming the indispensable role of GPX4 in the development of ferroptosis [162]. Multiple bypass pathways modulate I/R injury ferroptosis by altering GPX4 stability: the I/R‐accumulated metabolite 4‐HNE binds to GPX4 and OTUD5 to induce GPX4 K48‐linked ubiquitination and proteasomal degradation, whereas OTUD5 overexpression reverses ferroptotic damage [163]. Activation of the dsDNA–cGAS–STING axis also aggravates I/R injury by triggering the autophagic degradation of GPX4 [164]. Additionally, the inhibition of glutamine catabolism suppressed cardiomyocyte ferroptosis in isolated mouse I/R injury hearts, representing a metabolic bypass mechanism independent of direct GPX4 modulation [165]. These GPX4‐centered ferroptosis regulating strategies have been fully validated in preclinical models, supporting their translational value for I/R therapy. Lipid metabolism enzymes function as key auxiliary effectors that control the susceptibility to I/R injury ferroptosis. Arachidonate 15‐lipoxygenase‐1 (ALOX15) promotes lipid ROS generation by catalyzing phospholipid peroxidation. Cardiac‐specific ALOX15 knockout alleviates lipid peroxidation, ferroptosis, and I/R‐induced cardiac dysfunction, indicating a specific, role in promoting ferroptosis in the myocardium [166]. Targeted ALOX15 inhibition is a feasible preclinical strategy for reducing reperfusion‐related myocardial injury.

The NRF2/Hmox1 pathway is the canonical core signaling pathway that inhibits myocardial I/R injury ferroptosis. Mechanistically, NRF2 activation transcriptionally upregulates the expression of HO‐1, GPX4, and SLC7A11 while downregulating the expression of the proferroptotic ACSL4 and Drp1. This modulation mitigates mitochondrial damage and lipid peroxidation, reduces cardiomyocyte ferroptosis, and decreases myocardial infarct size [167, 168]. The involvement of this core antioxidant pathway has been consistently verified in multiple preclinical I/R injury models and serves as a reliable therapeutic target for I/R injury. Posttranslational modifications and epigenetic regulation of proteins mediate noncanonical bypass pathways to fine‐tune I/R injury ferroptosis. The adrenergic receptor agonist dexmedetomidine improves mitochondrial function and prevents ferroptosis by reducing lactic acid levels and mitigating the degree of malate dehydrogenase 2 (MDH2) lactylation, ultimately alleviating myocardial I/R injury [169]; thus, dexmedetomidine has high translational potential as a clinically available cardioprotective agent. N‐acetyltransferase 10 (NAT10) increases the stability of Mybbp1a by mediating ac4C acetylation, activating P53 and inhibiting SLC7A11 transcription. Moreover, P53, in turn, transcriptionally upregulates NAT10 expression, thereby exacerbating cardiac I/R injury [170]. In contrast, SUMO‐specific peptidase 2 (SENP2) promotes NCOA4 deSUMOylation, suppressing ferritinophagy‐dependent ferroptosis and mitigating I/R damage [171]. These relatively recently identified epigenetic and posttranslational regulatory mechanisms provide novel, preclinical targets for precisely managing I/R injury. Pharmacological evidence further confirms the pivotal role of ferroptosis in the pathogenesis of I/R injury. Preclinical studies have demonstrated that the ferroptosis inhibitor Fer‐1 and the iron chelators DFO/DXZ effectively alleviate myocardial damage in both acute and chronic I/R injury models [172, 173]. In summary, ferroptosis critically mediates myocardial I/R injury via the core NRF2/GPX4 pathways and multiple bypass regulatory axes. Targeted inhibition of cardiomyocyte ferroptosis has achieved robust preclinical efficacy and thus has promising translational prospects as a novel therapeutic strategy for clinical I/R injury.

#### 3.2.3 Ferroptosis and DOX‐Induced Cardiotoxicity

Despite its widespread use in treating tumors, DOX exhibits substantial cardiotoxicity, limiting its clinical application. DOX‐induced cardiomyocyte ferroptosis is predominantly driven by the disruption of cardiac iron homeostasis and inactivation of endogenous antioxidant signaling pathways, including the GPX4/NRF2 signaling pathways, which serves as the core regulatory axis governing ferroptotic processes in cardiomyocytes [172]. Iron overload and excessive lipid ROS accumulation are the primary initiating events of DOX‐related ferroptosis: DOX leads to significant upregulation of Hmox1 expression and the release of free iron from heme, resulting in increased free iron content and significantly increased lipid ROS concentrations in cardiomyocytes, thereby inducing ferroptosis [174].

The NRF2/GPX4 pathways jointly serve as the central antiferroptotic axis against DOX‐induced cardiotoxicity, which is finely regulated by multiple bypass molecules. E‐prostaglandin 1 receptor (EP1) protects cardiomyocytes from DOX‐induced ferroptosis by activating the PKC/NRF2 signaling pathway [175]. Hydrogen sulfide (H_2_S) inhibits DOX‐induced ferroptosis and cardiac injury by inducing KEAP1 S‐sulfhydration and activating the NRF2 antioxidant pathway [176]. The roles of both the EP1/PKC/NRF2 pathway and the H2S/KEAP1/NRF2 pathway have been fully validated in preclinical cellular and animal cardiotoxicity models, revealing robust translational potential as targets of cardioprotective interventions involving DOX chemotherapy. Conversely, protein arginine methyltransferase 4 (PRMT4) acts as a key negative regulator of the core NRF2/GPX4 axis: it exacerbates DOX‐induced cardiomyocyte ferroptosis by suppressing NRF2 activation and downstream GPX4 antioxidant signaling while accelerating lipid peroxidation and cardiac oxidative damage [177]. Targeted inhibition of PRMT4 reliably inhibited ferroptosis in preclinical myocardial injury models, suggesting a novel actionable target for alleviating chemotherapy‐induced cardiotoxicity.

Multiple tissue‐specific auxiliary regulatory molecules and bypass pathways further fine‐tune DOX‐induced cardiac ferroptosis independent of the core NRF2/GPX4 axis. Protosaponin A (PrA), an anti‐inflammatory compound extracted from hematoxylin, inhibits ACSL4 phosphorylation and subsequent phospholipid peroxidation by binding to ACSL4 and FTH1 while preventing autophagic degradation of the latter and subsequent Fe^2+^ release, thereby mitigating DOX‐induced ferroptosis and alleviating myocardial injury and dysfunction [178]. Given its natural derivation, small size, and low cytotoxicity, PrA has promising preclinical translational potential for the development of cardioprotective adjuvants to chemotherapy. Idebenone stabilizes FSP1 protein levels by inhibiting its ubiquitination–degradation, in turn inhibiting ferroptosis and alleviating DOX‐induced cardiotoxicity [179]. Idebenone is a clinically approved antioxidant drug that offers excellent translational advantages and rapid clinical transformation potential for repurposing as a chemotherapy‐related cardioprotectant.

Epigenetic regulators, including fat mass and obesity‐associated protein (FTO) and methyltransferase‐like 3 (METTL3), mediate the m6A‐dependent bypass regulation of cardiac ferroptosis with high cardiac‐tissue specificity. FTO inhibits DOX‐induced ferroptosis by activating P53‐P21/NRF2 in a HuR‐dependent m6A manner [180]. METTL3 catalyzes the m6A modification of TFRC mRNA, promoting DOX‐induced myocardial injury through an increase in cardiac iron uptake and induction of ferroptosis [181]. These m6A epigenetic regulatory mechanisms have only been verified in preclinical cardiac models, and thus further tissue‐specific validation is needed before they can be clinically translated.

Other bypass regulatory pathways involve multiple gene regulatory axes. The cellular repressor of e1a‐stimulated genes (CREG1) inhibits the mRNA and protein expression of pyruvate dehydrogenase kinase 4 (PDK4) by regulating the F‐box and WD repeat domain containing 7 (FBXW7)–forkhead box protein O1 (FOXO1) pathway, thereby inhibiting DOX‐induced ferroptosis [182]. Sorting nexin 3 (SNX3) promotes TFRC recycling through direct interactions, increasing iron accumulation and triggering ferroptosis and thus aggravating DOX‐induced myocardial injury [183]. Androgen‐induced gene 1 (AIG1) directly interacts with the E3 ubiquitin ligase Pirh2, promoting p53 ubiquitination–degradation and inhibiting DOX‐induced ferroptosis [184]. These newly identified regulatory axes are still being investigated through basic research; however, related preclinical data remain limited, and their translational prospects are undetermined.

In addition, mitochondria‐dependent ferroptosis plays a major role in DOX‐induced cardiotoxicity [185]. Mechanistically, DOX downregulates the expression of GPX4 and induces lipid peroxidation of the mitochondrial membrane through the complex formed by Fe^2+^ and DOX, leading to cardiomyocyte ferroptosis. However, overexpression of GPX4 or chelation of mitochondrial Fe^2+^ can inhibit DOX‐induced ferroptosis [185]. The mitochondrial outer membrane protein FUN14 domain containing 2 (FUNDC2) regulates mitochondrial GSH levels by interacting with the mitochondrial glutathione transporter SLC25A11 and participating in DOX‐induced myocardial ferroptosis [186], indicating that targeting mitochondria‐dependent ferroptosis may be important for inhibiting DOX‐induced cardiomyopathy. In summary, maintaining intracellular and intramitochondrial iron homeostasis and antioxidant systems may be a novel strategy for treating DOX‐induced ferroptosis and cardiomyopathy. Preclinical studies have verified that targeting mitochondrial iron overload and GPX4‐dependent antioxidant deficiency has significant cardioprotective effects, demonstrating the high translational potential of mitochondrial‐targeted adjuvant therapy for managing DOX cardiotoxicity.

In summary, the core network that regulates DOX‐induced cardiac ferroptosis primarily engages in NRF2/GPX4‐mediated antioxidant defense and iron homeostasis maintenance, while multiple bypass signaling, epigenetic, mitochondrial, and metabolic pathways serve as auxiliary regulatory mechanisms. Preclinical evidence has fully validated the therapeutic efficacy of these core and bypass targets, indicating their broad translational potential for the clinical prevention and treatment of DOX‐induced cardiac complications.

#### 3.2.4 Ferroptosis and HF

HF is a common clinical cardiovascular syndrome characterized by myocardial hypertrophy and myocardial fibrosis. Disrupted myocardial iron homeostasis and excessive lipid peroxidation form the core effector machinery of HF‐related ferroptosis, involving the NRF2/GPX4 pathways, Toll‐like receptor 4 (TLR4)/NOX4‐mediated oxidative stress, NCOA4‐dependent ferritin autophagy, and microbial metabolite‐related cascades as the core signaling axis. Clinical studies have shown that both iron deficiency and overload can induce HF through their role in myocardial iron homeostasis [187, 188, 189]. Targeting ferroptosis and iron balance has emerged as a promising therapeutic direction for patients with HF. Ferritin deficiency impairs intracellular iron storage, causing excessive ROS accumulation and accelerating age‐associated cardiac injury [190]. In HF, NCOA4‐mediated ferritinophagy upregulates the expression of mitochondrial iron transporter sideroflexin1 (SFXN1), inducing mitochondrial iron overload, ROS overproduction, and severe lipid peroxidation, ultimately initiating cardiomyocyte ferroptosis [191]. The NCOA4/SFXN1 mitochondrial ferroptosis pathway is a cardiac‐specific regulatory axis, and pharmacological inhibition of the associated ferritinophagy has been suggested as a feasible, preclinical strategy for treating HF.

Core oxidative stress signaling regulators play key roles in ferroptotic cardiac injury in HF. Bioinformatic analysis of cardiac tissue revealed that the expressions of TLR4 and NADPH oxidase 4 (NOX4) are significantly upregulated in HF tissue, but TLR4 or NOX4 knockdown can ameliorate HF symptoms by inhibiting cardiomyocyte autophagy and ferroptosis [192]. In a pressure overload‐induced HF model, puerarin increased the expression of GPX4 while inhibiting that of NOX4, thereby mitigating lipid peroxidation and ferroptosis, improving the myocardial microstructure and mitigating mitochondrial damage [193]. The importance of the TLR4/NOX4/GPX4 cascade has been well validated in multiple HF models, while puerarin represents a promising natural ferroptosis‐targeted agent in adjuvant therapy for HF.

Pharmacological and microbial bypass regulators modulate HF ferroptosis independent of the core iron signaling pathway. LuQi formula (LQF) mitigates HF injury and represses cardiomyocyte ferroptosis by activating the NRF2/GPX4 pathways and downregulating SLC7A11 and HO‐1 expression [194]. A safe and multitargeted herbal formulation, LQF has favorable translational prospects for HF intervention. Additionally, *Faecalibacterium prausnitzii*‐derived butyrate suppresses cardiomyocyte lipocalin‐2 (LCN2) expression, reduces iron deposition, and inhibits ferroptosis, thereby slowing age‐related HF [195]. This gut–heart regulatory mechanism provides an innovative and noninvasive target for preventing HF with promising clinical transformational value. In addition, ACSL4‐dependent ferroptosis activates the pyroptosis signaling pathway, leading to increased production of the proinflammatory cytokine IL‐1β, which in turn promotes HF [196], indicating that multiple methods of cell death interact to jointly regulate HF‐induced myocardial damage. Therefore, ferroptosis plays important pathophysiological roles in the development and progression of HF, but a more comprehensive understanding of the mechanisms underlying these roles is necessary for further exploring effective targets for improving the outcomes of HF patients.

#### 3.2.5 Ferroptosis and Hypertrophic Cardiomyopathy

Hypertrophic cardiomyopathy (HCM) is a genetic myocardial disease characterized by asymmetric ventricular hypertrophy. Studies have shown that animal models of HCM induced by multiple methods, including pressure overload, angiotensin II (Ang II) infusion, transverse aortic constriction (TAC), and isoproterenol administration, are accompanied by certain features of ferroptosis, such as excessive accumulation of lipid peroxidation, suggesting that ferroptosis may be involved in the pathological processes leading to HCM [197, 198]. The core mechanism by which ferroptosis influences the development of HCM involves SLC7A11/GPX4 antioxidant signaling, iron metabolism‐dependent ferroptosis itself, and multiple stress‐related bypass pathways, which synergistically drive HCM development and myocardial remodeling. Iron metabolism‐related effector molecules directly trigger ferroptosis to facilitate HCM. A deficiency in ferritin impairs cardiac iron storage, while a high‐iron diet induced severe ferroptotic injury and typical HCM phenotypes in mice [199]. NCOA4‐mediated ferritinophagy acts as a key effector mechanism in TAC‐induced HCM, whereas inhibition of NCOA4 effectively suppresses ferroptosis and alleviates cardiac remodeling [200]. Additionally, PM2.5 exposure induces ferritin autophagy, causing intracellular and mitochondrial iron deposition, mitochondrial dysfunction and lipid peroxidation, thereby triggering cardiomyocyte ferroptosis and myocardial hypertrophy [201].

Core antioxidant signaling molecules regulate the progression of HCM progression via redox modulation. In aortic banding and isoproterenol‐induced HCM models, NOX4 upregulation suppressed GPX4 activity, triggering excessive ROS production and cardiomyocyte ferroptosis to induce myocardial hypertrophy and remodeling [193]. Elevated levels of mixed lineage kinase 3 (MLK3) lead to a decrease in GPX4 expression, ultimately resulting in ferroptosis and exacerbating TAC‐induced HCM [202]. Genetic deletion of the ferroptosis regulator SLC7A11 also exacerbates Ang II‐induced myocardial fibrosis, myocardial hypertrophy, and cardiac dysfunction [199]. In addition, protein kinase D (PKD) knockdown relieves Ang II‐induced myocardial hypertrophy and ferroptosis by impairing the JNK/P53 cascade [203]. Targeted NOX4 inhibition and GPX4 activation have been fully validated as potential therapeutic modalities in preclinical HCM models, supporting the reliable translational potential of these methods for targeted HCM therapy.

In summary, ferroptosis plays an important role in HCM, but the field is still in its infancy. Current evidence indicates that targeting core effector (GPX4 and SLC7A11) or upstream signaling molecules (NOX4, NCOA4, and MLK3) holds promise for managing HCM. However, the evidence for most interventions remains preclinical; future studies should prioritize cardiac‐specific delivery systems, the repurposing of clinically available iron chelators or NOX4 inhibitors, and rigorous testing in genetic HCM models. Therefore, further exploration of the regulatory mechanisms that underlie the role of ferroptosis in HCM is warranted to identify effective therapeutic targets.

#### 3.2.6 Ferroptosis and Atherosclerosis

Atherosclerosis (AS) progression is predominantly driven by endothelial dysfunction, macrophage foam cell formation, and vascular inflammation, in which ferroptosis acts as a central pathogenic mediator [204]. The NRF2/GPX4 antioxidant axis serves as the core protective signaling module that regulates vascular ferroptosis. Iron transporters (DMT1 and SCARB1) and the ferritin autophagy effector NCOA4 function as core effector molecules that trigger iron overload and lipid peroxidation. Multiple cytokines, receptors, and transcription factors (LOX‐1, STAT6, YAP1, and Gsα), meanwhile, act as tissue‐specific bypass regulator molecules to fine‐tune vascular cell ferroptosis, endothelial injury, and plaque progression.

The expression of core NRF2/GPX4 antioxidant signaling regulators determines the induction of vascular ferroptosis and plaque stability. Micheliolide (MCL) suppresses macrophage ferroptosis by influencing the formation of the KEAP1/NRF2 complex, thereby alleviating AS [205]. A natural antioxidant compound with high biosafety, MCL has promising potential for the development of novel anti‐AS ferroptosis‐targeted agents. Endothelial stimulatory G protein α (Gsα) subunit deficiency transcriptionally impairs NRF2 signaling activity to facilitate endothelial ferroptosis and aggravate AS [206]. Conversely, oxidized low‐density lipoprotein (ox‐LDL) initiates endothelial ferroptosis by suppressing GPX4 activity [207], a classic upstream trigger of endothelial dysfunction and early AS lesion formation. The ferroptosis inhibitor Fe‐1 increases the concentrations of FTH, GPX4, and SCARB1 by activating AMPK; reduces the iron concentration and lipid accumulation in macrophages treated with ox‐LDL; and alleviates AS and foam cell formation [208]. YAP1 promotes glutamate and glutathione synthesis by regulating glutaminase 1 (GLS1) expression and inhibits ferroptosis in vascular smooth muscle cells [209]. This GPX4‐dependent mechanism has been widely validated in ox‐LDL‐induced vascular injury models, providing a reliable preclinical target for early AS prevention.

Core iron metabolism‐related effector molecules directly drive vascular ferroptosis and plaque progression. LOX‐1 activates the cGAS–STING pathway to upregulate the expression of the ferritin autophagy receptor NCOA4 and promotes the interaction between STING and NCOA4 to drive ferritin autophagy and potentiate lipid peroxidation, exacerbating aortic endothelial inflammation and AS [210]. In contrast, STAT6 inhibits endothelial ferroptosis and AS progression by transcriptionally downregulating DMT1 and SOCS1 expression [211]. These iron‐metabolic and transcriptional regulatory axes function specifically in vascular tissues and thus offer precision targets for vascular‐specific antiferroptosis therapy.

Bypass regulatory molecules and cytokine‐mediated pathways mediate noncanonical ferroptosis in AS. N‐acetylneuraminic acid (Neu5Ac) promotes SLC3A2‐related endothelial ferroptosis to trigger endothelial injury and accelerate plaque progression [212]. Recombinant interleukin 17D (IL‐17D) induces ferroptosis through the CD93/miR‐181a‐5p/SLC7A11 signaling pathway and promotes endothelial cell inflammation, thereby accelerating AS [213]. Fe‐1 and YAP1‐mediated pathways are protective and highly efficacious in preclinical AS models, supporting their great potential for future targeted drug development. In conclusion, the development of therapeutic drugs that address ferroptosis could result in benefits for AS patients, but more in‐depth research on the roles of ferroptosis are needed.

### 3.3 Neurodegenerative Disorders

NDDs are a group of progressive conditions characterized by gradual neuronal loss and deterioration of neural network integrity; classic examples include Alzheimer's disease (AD), Parkinson disease (PD), amyotrophic lateral sclerosis (ALS), and multiple sclerosis (MS) [214]. Owing to the limited regenerative capacity of postmitotic neurons, structural and metabolic disturbances can lead to irreversible cognitive, motor, and behavioral impairments, which have resulted in profound socioeconomic and health care burdens [215]. Recent studies have demonstrated that iron dyshomeostasis, lipid peroxidation, and oxidative stress are common pathological features in multiple NDDs, suggesting that ferroptosis may serve as a critical link among neuroinflammation, mitochondrial dysfunction, and neuronal loss [216]. However, the upstream triggers and regulatory networks governing ferroptosis in these contexts vary among different disorders. In AD, ferroptosis is closely associated with β‐amyloid (Aβ) deposition and tau pathology; in PD, it is linked to nigral iron accumulation and aberrant microglial activation; and in ALS, the susceptibility to ferroptosis is more closely related to impaired iron homeostasis in motor neurons and RNA‐binding protein dysfunction. Therefore, elucidating disease‐specific ferroptotic mechanisms may facilitate the development of targeted therapeutic strategies for NDDs.

#### 3.3.1 Alzheimer's Disease

AD is a progressive NDD characterized by Aβ plaque deposition, tau hyperphosphorylation, synaptic dysfunction, and neuronal loss [217, 218]. Emerging evidence indicates that iron dyshomeostasis is a key pathological hallmark of AD: excessive iron accumulation facilitates lipid peroxidation and neuronal damage, whereas aberrant iron metabolism is closely associated with mitochondrial dysfunction, neuroinflammation, and progressive cognitive impairment [15, 219]. Notably, in AD, ferroptosis is closely linked to impaired energy metabolism. Mitochondrial dysfunction results in ATP depletion, which limits glutathione (GSH) synthesis and weakens neuronal antioxidant defenses, thereby increasing susceptibility to ferroptosis [220]. Furthermore, NOX4‐driven mitochondrial metabolic dysfunction has been shown to promote mtROS accumulation and ferroptosis in astrocytes [221]. Consistent with these findings, disruption of NRF2 signaling aggravates oxidative stress and mitochondrial injury in astrocytes, thereby increasing neuronal vulnerability in AD [222]. These findings indicate that alterations in energy metabolism, oxidative stress, and antioxidant defense jointly contribute to the molecular mechanisms by which ferroptosis influences the pathogenesis of AD, providing potential therapeutic targets for regulating the relevant metabolic processes and inhibiting ferroptosis.

#### 3.3.2 Parkinson's Disease

PD is a chronic NDD characterized by the progressive loss of dopaminergic neurons in the substantia nigra and abnormal aggregation of α‐synuclein, leading to the formation of Lewy bodies and neurites. Exhibiting the second‐highest prevalence among NDDs after AD, PD clinically presents with resting tremors, bradykinesia, rigidity, and a wide spectrum of nonmotor symptoms, leading to substantial disability and reduced quality of life. More than 6 million individuals worldwide are currently affected, and its prevalence is projected to double over the next three decades, potentially making PD a major cause of neurological disability [223, 224, 225]. Studies have shown that the development of PD is associated with disrupted iron homeostasis, oxidative stress, and mitochondrial dysfunction, among which abnormal iron accumulation is considered a critical factor that contributes to the neuronal damage and progression of the disease [226, 227]. Moreover, microglia exhibit marked iron accumulation and inflammatory activation during the course of PD; however, how iron overload affects microglia function and whether it contributes to ferroptosis‐mediated neuronal injury remain unclear [228, 229]. Therefore, elucidating the role of microglial iron dysregulation and ferroptosis in PD is highly important for understanding the pathogenesis of the disease and identifying novel therapeutic targets. Johnson et al. employed a human‐induced pluripotent stem cell‐derived triculture system to investigate the effects of iron dysregulation on microglial function and neurodegeneration. Those authors revealed that microglia exhibited a pronounced transcriptional response to iron imbalance and developed a ferroptosis‐associated signature subset of genes whose expression is elevated in the midbrains of patients with PD. Iron overload‐induced microglial ferroptosis exacerbated neuronal injury by promoting lipid peroxidation and oxidative stress, whereas microglial depletion attenuated neurotoxicity. Genome‐wide CRISPR screening revealed that SEC24B is a novel regulator of ferroptosis, underscoring the important role of microglial iron overload and ferroptosis in the pathogenesis of PD [230]. Furthermore, upon toxic stimulation, microglial complement receptor CR3 is activated and drives assembly of the NOX2 complex, resulting in the production of large amounts of ROS. These ROS diffuse and subsequently act on adjacent dopaminergic neurons, reprogramming their iron metabolism: the expressions of TfR and DMT1 are upregulated, while those of FPN1 and ferritin are downregulated, leading to expansion of the labile iron pool. Iron overload and oxidative stress synergistically induce lipid peroxidation and decrease GPX4 expression, triggering neuronal ferroptosis and accelerating nigral dopaminergic degeneration. Similarly, CR3 blockade/knockout or NOX2 inhibition reduces iron deposition and lipid peroxidation, improving outcomes at the tissue and behavioral level. Exogenous iron supplementation can partially reverse this protective effect, confirming the iron‐dependent nature of this pathway [231]. Recent work has indicated that voluntary wheel running exerts a protective effect on PD pathology, partly through the modulation of microglial ferroptosis. Exercise increases microglial SLC7A11 expression and promotes the interaction of this molecule with ALOX12, decreasing the activity of the latter and thereby restoring iron homeostasis, reducing lipid peroxidation, and mitigating mitochondrial ferroptotic injury. These coordinated effects collectively mitigate microglial ferroptosis and contribute to improved neuroinflammatory and neurodegenerative outcomes in PD models [232].

#### 3.3.3 Lateral Sclerosis Ferroptosis

ALS, the most common adult‐onset motor neuron disease, is characterized by progressive degeneration of upper motor neurons in the motor cortex and lower motor neurons in the brainstem and spinal cord, accompanied by pathological aggregation of proteins such as TAR DNA binding protein (TDP‐43) or FUS RNA binding protein (FUS). Clinically, patients with ALS exhibit pronounced symptom heterogeneity, typically presenting with limb‐onset or bulbar‐onset weakness and eventually progressing to respiratory failure, with a median survival of only 2–5 years [233]. The global prevalence of ALS is approximately 4–5 per 100,000 individuals, and the risk is higher in men; moreover, up to half of patients experience cognitive and/or behavioral impairment, further compounding the burden of the disease [234, 235]. The pathogenesis of ALS involves multiple converging processes, including RNA dysmetabolism, disrupted proteostasis, mitochondrial dysfunction, and neuroinflammation, while increasing evidence implicates dysregulated iron homeostasis and lipid peroxidation as key determinants of motor neuron vulnerability, suggesting that ferroptosis may be a potential driver of neurodegeneration in individuals with ALS [236, 237, 238]. Sustained depletion of the ferroptosis‐suppressing enzyme GPX4, together with disrupted iron homeostasis, drives pathological lipid peroxidation and underlies the selective vulnerability of motor neurons in ALS. Restoring GPX4 activity effectively stops the ferroptotic cascade, improves neuronal survival, and mitigates disease progression, highlighting the pathogenic and therapeutic relevance of ferroptosis in ALS [239]. More recently, SPY1 has been identified as an upstream modulator of ferroptotic susceptibility. ALS models have demonstrated MDM2‐dependent ubiquitination degradation of SPY1, resulting in impaired activation of the GCH1–BH4 antioxidant axis and insufficient repression of TFR1‐mediated iron uptake. This dual disruption promotes iron accumulation and pathological lipid peroxidation, whereas neuron‐targeted restoration of SPY1 normalizes these processes, improves mitochondrial integrity, and delays disease onset and progression [240]. Although increasing evidence supports the involvement of ferroptosis in the pathogenesis of ALS, studies remain lacking relative to those on AD and PD. Moreover, given the substantial clinical and genetic heterogeneity of the disorder and the lack of experimental models that faithfully recapitulate human pathology, few investigations have explored the mechanistic basis by which ferroptosis influences ALS development. Future studies integrating single‐cell omics, spatial transcriptomics, and patient‐derived models are needed to define the cell type‐specific roles and therapeutic potential of ferroptosis in ALS.

### 3.4 Other Pathological Contexts

#### 3.4.1 Chronic Kidney Disease

Kidney diseases have been increasingly identified as a major challenge in global public health. Over the past decades, mortality attributable to these disorders has continued to increase, outpacing that of many other prevalent chronic conditions. Epidemiological projections further indicate that by 2040, chronic kidney disease (CKD) will become the fifth leading cause of death worldwide [241, 242, 243, 244]. CKD is characterized by diverse etiologies, including diabetes, hypertension, glomerulonephritis, and nephrotoxin exposure. Current management only slows functional decline and mitigates major complications but remains insufficient for preventing progression to end‐stage renal disease. Given the rising global burden, the identification of new molecular targets and the development of mechanism‐based therapeutic strategies are urgent [242, 245]. The pathological relevance of ferroptosis in CKD has attracted increasing attention [246]. Existing studies have focused on determining the presence of ferroptosis in CKD models and patient tissues, elucidating the molecular mechanisms underlying ferroptosis‐mediated renal injury, and identifying key regulators that influence progression in CKD. These insights offer valuable guidance for understanding the pathogenesis of CKD and developing new therapeutic targets for the condition. Diabetic nephropathy (DN), the most common microvascular complication of diabetes and a leading cause of end‐stage renal failure, remains inadequately managed despite the number of existing therapies. Increasing evidence indicates that tubular injury often precedes glomerular alterations; for example, mitochondrial dysfunction in proximal tubular epithelial cells (PTECs) and PTEC‐derived metabolites can impair podocyte function. Moreover, tubular atrophy and interstitial fibrosis, mediated by inflammatory and profibrotic signaling, accelerate renal functional decline. These findings underscore the pivotal role of tubular pathology in the progression of DN. Recent evidence has indicated that vitamin D receptor (VDR) confers significant renoprotection in DN by modulating the Nrf2/HO‐1 signaling cascade to limit tubular ferroptosis. Hyperglycemic conditions suppress VDR–NRF2 activity, resulting in disrupted iron homeostasis and increased oxidative stress, thereby precipitating ferroptotic injury within proximal tubular cells. Pharmacologic activation of VDR restores Nrf2/HO‐1‐dependent antioxidant and iron‐regulatory pathways and increases the expression of key ferroptosis‐related molecules such as GPX4 and SLC7A11, ultimately attenuating tubular damage and slowing disease progression [247]. Moreover, in DN, canopy FGF signaling regulator 2 (CNPY2) promotes tubular ferroptosis through activation of the p‐ERK/ATF4/ChaC glutathione‐specific gamma‐glutamylcyclotransferase 1 (CHAC1) pathway, thereby impairing cellular antioxidant defenses and the accompanying MAM disruption and mitochondrial dysfunction. Conversely, inhibition of CNPY2 expression ameliorates oxidative stress and iron dysregulation, ultimately mitigating tubular injury [248]. Metabolic dysregulation plays a central role in tubular injury during diabetic kidney disease (DKD). The accumulation of lactate in this disease induces p300‐mediated lactylation of tripartite motif containing 65 (TRIM65) at lysine 206, thereby impairing its E3 ubiquitin ligation and leading to the aberrant accumulation of iron responsive element binding protein 2 (IREB2) and PDK4. Elevated IREB2 increases iron uptake and lipid peroxidation, triggering ferroptosis, whereas increased PDK4 expression suppresses mitochondrial pyruvate oxidation, drives glycolytic reprogramming, and further exacerbates lactate buildup. These two pathways synergistically amplify tubular injury and form a vicious metabolism–ferroptosis cycle, while restoring TRIM65 activity effectively attenuates both ferroptosis and metabolic imbalance, ultimately mitigating DKD progression [249]. These findings underscore the central role of ferroptosis in tubular injury during DKD and identify actionable molecular pathways for therapeutic intervention. The mechanisms elucidated to date offer valuable insights for improving clinical outcomes and reducing the global burden of DKD.

#### 3.4.2 Acute Kidney Injury

Acute kidney injury (AKI) is defined as an abrupt decline in renal function caused by a variety of insults such as ischemia, hypoxia, infection, sepsis, metabolic imbalance, obstruction, and nephrotoxicity. In the short term, AKI leads to fluid overload, electrolyte and acid–base disturbances, and immune dysfunction; over time, it markedly increases the risk of CKD, kidney failure, and mortality. Because full renal recovery is relatively unlikely, many patients remain susceptible to functional decline [250]. Tubular epithelial cells (TECs), whose functions depend on mitochondrial oxidative phosphorylation, are highly susceptible to ischemic, oxidative, and metabolic stress. Typical histopathological features of AKI include brush‐border loss, epithelial flattening and detachment, inflammatory infiltration, and Tamm–Horsfall protein‐rich casts. TECs display notable plasticity in AKI: mild injuries can be repaired through dedifferentiation, migration, proliferation, and redifferentiation, whereas severe or repeated insults can lead to maladaptive repair with structural damage, sustained inflammation, and fibrosis [251]. Owing to the phenotypic diversity of and dynamic transitions among TECs, the mechanisms responsible for their fate after AKI remain incompletely defined. In this context, ferroptosis, characterized by iron‐dependent lipid peroxidation, is considered a core mechanism that drives TEC injury. In cisplatin‐induced AKI, mitochondrial injury, ROS accumulation, and disrupted iron handling synergistically destabilize the HO‐1/GPX4 axis and trigger tubular ferroptosis. BNIP3– and PINK1–PARK2‐dependent mitophagy removes dysfunctional mitochondria, decreases oxidative and iron stress, and maintains GPX4 activity, thereby mitigating ferroptosis. Impaired mitophagy increases mitochondrial damage and ferroptotic signaling, highlighting its indispensable protective role against cisplatin nephrotoxicity [252]. Moreover, cisplatin exacerbates tubular injury by disrupting mitochondrial dynamics. Cisplatin‐induced phosphorylation of pyruvate kinase m2 (PKM2) at Tyr105 promotes its transition from its tetrameric form to its dimeric form and facilitates its translocation to mitochondria, where it interacts with MYH9 to activate Drp1 and drive mitochondrial fragmentation. This mitochondrial fission event precipitates multiple forms of tubular cell death, including apoptosis, necroptosis, and ferroptosis, thereby accelerating AKI progression. Pharmacologic inhibition of PKM2 or blockade of its mitochondrial translocation preserves mitochondrial integrity and mitigates cisplatin‐induced nephrotoxicity [253]. Emerging evidence further indicates the importance of targeting upstream oxidative stress in AKI. Remote ischemic preconditioning (rIPC) attenuates injury by suppressing the NOX4‐derived ROS axis in cisplatin‐, LPS‐, and IRI‐induced AKI, restoring mitochondrial dynamics and mitophagic flux while normalizing GPX4 and ACSL4 expression to limit ferroptosis. Importantly, NOX4 overexpression negates, whereas NOX4 deficiency potentiates, the renoprotective actions of rIPC, underscoring the role of NOX4 as a pivotal regulator of its efficacy [254]. Notably, AKI is not a self‐limited acute pathological event. If ferroptotic damage to renal TECs fails to be effectively suppressed in the acute phase, persistent lipid peroxidation will trigger chronic inflammatory infiltration and excessive extracellular matrix deposition, gradually inducing renal interstitial fibrosis and eventually driving the progression from transient acute renal dysfunction to irreversible CKD. In this process, sustained ferroptosis serves as a crucial molecular bridge connecting acute and chronic renal injury. In summary, ferroptosis plays a central role in AKI and may contribute to its progression to CKD. Given its involvement in tubular injury, inflammation, and fibrogenesis, ferroptosis represents a promising therapeutic target for preventing acute kidney damage and delaying the progression to chronic renal disease.

#### 3.4.3 Acute Liver Injury

Acute liver injury (ALI) is characterized by rapid hepatocellular damage from a variety of insults, including drug toxicity, viral infection, I/R injury, and those caused by autoimmune disorders [255]. In severe cases, ALI can progress to acute liver failure, which is associated with high mortality. Drug‐induced liver injury, particularly acetaminophen (APAP) overdose, is among the most common causes of ALI. Despite substantial advances in the understanding of the pathogenesis of ALI, effective therapeutic options remain limited. In recent years, ferroptosis, an iron‐dependent form of RCD driven by lipid peroxidation, has been identified as an important contributor to this pathogenesis. APAP overdose induces glutathione depletion, iron dyshomeostasis, and mitochondrial dysfunction. Under physiological conditions, APAP‐induced hepatocyte death is predominantly mediated by mitochondrial oxidative stress and mitochondrial permeability transition. However, under conditions of iron overload or compromised antioxidant defense, lipid peroxidation increases, suggesting the involvement of ferroptotic signaling in the progression of liver injury [256].

Disruption of GSH metabolism is considered a critical link between APAP toxicity and ferroptosis. The concentration of ChaC glutathione‐specific gamma‐glutamylcyclotransferase 1 (CHAC1), a glutathione‐degrading enzyme, is markedly increased during APAP‐induced liver injury. Increased CHAC1 concentrations further deplete intracellular GSH pools, reduce protein S‐glutathionylation, and promote TFRC‐mediated iron uptake, thereby exacerbating ferroptotic cell death. In contrast, CHAC1 deficiency preserves redox homeostasis, attenuates hepatocyte ferroptosis, and heals liver injury [257]. The Nrf2 antioxidant pathway represents a major defense mechanism against ferroptosis. Nrf2 activation increases GPX4 expression and antioxidant activity, thereby limiting lipid peroxidation. Pharmacological activation of Nrf2 with kaempferol attenuates APAP‐induced iron accumulation, oxidative stress, and ferroptotic damage [258]. Similarly, the use of mifepristone (RU486) inhibits ferroptosis through NRF2 activation and facilitates the detoxification of lipid peroxidation products such as 4‐HNE, ultimately protecting against APAP‐induced liver injury [259]. In addition to intracellular regulatory pathways, gut microbiota‐derived metabolites also modulate APAP‐associated ferroptosis. *Lactobacillus vaginalis* produces β‐galactosidase, which produces daidzein from dietary isoflavone precursors. Daidzein, in turn, suppresses the expression of farnesyl diphosphate synthase expression and activates the Nrf2 signaling axis, thereby reducing lipid peroxidation and ferroptosis and ultimately attenuating APAP‐induced liver injury [260]. In summary, APAP‐induced glutathione depletion and redox imbalance are pivotal triggers of ferroptosis. Dysregulated iron homeostasis, lipid peroxidation, and defective antioxidant defenses act coordinately to drive ferroptotic liver injury. Therapeutic interventions targeting these ferroptosis‐associated pathways may offer promising approaches for the management of ALI.

Overall, the information summarized in this chapter reveals that ferroptosis acts as a critical and pervasive pathogenic driver that underlies multiple human diseases via distinct pathological mechanisms and functional patterns. Recent studies have demonstrated prominent cell type‐specific differences in ferroptosis sensitivity, which are governed to distinct degrees by iron homeostasis, lipid metabolism and antioxidant capacity, ultimately defining the tissue‐specific pathological roles and therapeutic potential of ferroptosis. In terms of iron regulation, renal TECs typically highly express TfR1 while poorly expressing FPN, and thus cytosolic Fe^2+^ is prone to accumulation and subsequent amplification of ROS generation via the Fenton reaction [261, 262]. In contrast, ferroptosis‐resistant cells such as hepatocytes and neurons maintain stable iron pools through robust iron chelation and efflux mechanisms [263]. Furthermore, differential subcellular iron distribution exacerbates cellular vulnerability: mitochondrial iron overload is frequently observed in cardiomyocytes, whereas lysosomal iron release plays a pivotal role in macrophage ferroptosis. Lipid metabolism also modulates ferroptosis sensitivity: M2 macrophages highly express key enzymes involved in lipid metabolism, including ACSL4 and LPCAT3, facilitating the enrichment of PUFA‐PLs and thereby providing abundant substrates for lipid peroxidation [264]; conversely, resistant cells, such as S100P‐overexpressing HCC cells, suppress PUFA‐PL synthesis by promoting lysosomal degradation of ACC1 and other rate‐limiting enzymes, thereby reducing susceptibility to lipid peroxidation [265]. Heterogeneity in antioxidant capacity is another critical determinant of ferroptosis sensitivity. CD8^+^T cells within the tumor microenvironment are prone to ferroptosis because they compete for cystine and have insufficient GPX4 activity [266, 267], whereas melanoma cells acquire substantial resistance through GPX4 palmitoylation [268]. Collectively, these cell type‐specific ferroptosis mechanisms underlie the diverse pathological manifestations of oxidative stress, metabolic dysfunction and immune dysregulation across various tissues and diseases. Clarifying these heterogeneous regulatory characteristics further would provide critical insights for developing precise, targeted ferroptosis‐based therapeutic strategies for multiple human diseases.

## 4 Targeting Ferroptosis: Diagnostic and Therapeutic Strategies

Ferroptosis is an iron‐dependent, lipid peroxidation‐driven form of cell death that plays key roles in disease pathogenesis and therapeutic responses and thus represents a viable target for precise disease intervention. However, the clinical translation of ferroptosis‐targeted therapy is hindered by poor agent selectivity, insufficient delivery efficiency, a lack of consistent diagnostic biomarkers, and context‐dependent therapeutic heterogeneity. This chapter seeks to systematically illustrate the current progress and core challenges in ferroptosis‐targeted diagnosis and treatment: it summarizes pharmacological modulation via representative ferroptosis inducers and inhibitors, followed by existing nanotechnology‐based targeted delivery strategies and up‐to‐date information on histochemical and circulating biomarkers for in vivo ferroptosis detection. Finally, the critical limitations of context‐dependent effects and narrow therapeutic windows are discussed, aiming to provide insights for optimizing and translating ferroptosis‐targeted therapeutic regimens.

### 4.1 Pharmacological Modulation: Ferroptosis Inducers and Inhibitors

Increasing evidence on the mechanisms of action of ferroptosis has identified many specific inducers and inhibitors of this form of cell death [269]. These agents not only serve as valuable tools for elucidating the mechanisms underlying ferroptosis but also offer promising therapeutic targets for diseases such as cancer and NDDs. Ferroptosis inducers have demonstrated significant potential in oncological therapy through the targeting of key cellular processes—including iron metabolism, lipid peroxidation, and antioxidant defense systems—that promote ferroptosis, especially in tumor cells that are resistant to conventional chemotherapy or refractory to radiotherapy. Conversely, ferroptosis inhibitors protect cells from ferroptosis by targeting key ferroptosis pathways, protecting against neurodegenerative diseases and I/R injury. For ease of comprehension, the core drug and compound categories (e.g., GPX4 inhibitors and iron chelators), mechanisms, and therapeutic applications of these modulators are compiled in Table 2. Furthermore, we summarize the key aspects of preclinical and clinical studies that targeted the ferroptosis pathway in human disease, including disease models, drug interventions, mechanisms, and research phases, in Table 3.

**TABLE 2:** The main inducers and inhibitors of ferroptosis.

Types	Mechanisms	Drugs and compounds	Application	References
Inducer	Inhibit system Xc^−^ and prevent cystine import	Sorafenib, sulfasalazine Erastin, IKE, APAP, PE	Cancer therapy	[7, 67, 270, 271, 272, 273]
	Inhibit GPX4	Withaferin A, gemcitabine, RSL3, ML162, ML210, FIN56, FINO2	Cancer therapy	[67, 274, 275, 276, 277, 278]
	Inhibition of GCL; GSH‐depleting	BSO, AUF, DP12, cisplatin, acetaminophen, cyst(e)inase	Cancer therapy	[50, 279, 280]
	Iron homeostasis imbalance	Ferric citrate, lapatinib, artesunate, hemin, FINO_2_	Cancer therapy	[278, 281, 282, 283, 284]
Inhibitor	Iron chelator	Neratinib, artesunate, CPX, DFO, DFP, DFX, DXZ	Neuroprotection, organ injury, myocardial protection	[172, 285, 286, 287, 288, 289, 290]
	Inhibit lipid peroxidation	Ferrostatin‐1, liproxstatin‐1, vitamin E, BHT, BHA, CoQ_10_, idebenone, baicalein, vildagliptin, troglitazone, rosiglitazone, zileuton, 2‐acetylphenothiazine, GKT137831, AA‐861	Neuroprotection, myocardial protection	[5, 67, 68, 291, 292, 293, 294, 295, 296, 297]
	Activate system Xc^−^	Cycloheximide	Neuroprotection, organ injury	[298]

*Note*: The main inducers and inhibitors of ferroptosis.

Abbreviations: IKE, imidazole ketone erastin; APAP, acetaminophen; PE, phosphatidylerhanolamine; RSL3, 1S,3R; DPI7/10, diphenyleneiodonium chloride 7/10; BSO, buthionine sulfoximine; AUF, auranofin; CPX, ciclopirox; DFO, deferoxamine; DFP, deferiprone; DFX, deferasirox; DXZ, dexrazoxane; BHT, butylated hydroxytoluene; BHA, butylated hydroxyanisole; CoQ_10_, coenzyme Q_10_.

**TABLE 3:** Summarize the key aspects of preclinical and clinical studies targeting ferroptosis pathway in human disease.

Type of disease	Disease	Model/crowd	Intervention target/drug	Target mechanism of ferroptosis	Key outcomes (preclinical)	Key outcomes (clinical)	Phase	References
Cardiovascular diseases	Ischemia–reperfusion (I/R) injury	Mouse myocardial tissues	Fer‐1	Inhibition of lipid peroxidation	Reduce myocardial infarct size, improve mitochondrial function	N/A	Preclinical	[172]
	Diabetic cardiomyopathy	Mouse myocardial tissues	Nicorandil	Inhibition of ACSL4‐mediated mitochondrial lipid peroxidation	Activation of Parkin‐dependent mitophagy suppresses mitochondrial translocation of ACSL4 and ferroptosis.	N/A	Preclinical	[299]
	DOX‐induced cardiotoxicity	Mouse myocardial tissues	BRD4770	Reduce the production of reactive oxygen species (ROS) and lipid peroxidation, and maintain glutathione homeostasis	Promote the NRF2/ATF4–SLC7A11 axis	N/A	Preclinical	[300]
Neurodegenerative diseases	Ischemic stroke	Patients	DFO	Iron chelation and antioxidation	Antioxidation	Partial benefit	Phase II clinical trial	[301]
	Alzheimer's disease	Transgenic mice	Thonningianin A	Activate GPX4 via the AMPK/Nrf2 pathway	Alleviate neuronal damage	N/A	Preclinical	[302]
	Amyotrophic lateral sclerosis	Patients	PTC857	Suppresses 15‐LO activity to alleviate oxidative stress and preserve glutathione levels	Ameliorate neurodegeneration	Partial benefit	Phase II clinical trial	[303]
Cancers	Colorectal cancer	Patients	Sorafenib	Inhibit SLC7A11 expression and promote ferroptosis	Overcome radiotherapy resistance	Partial benefit	Phase II clinical trial	[304]
	Acute myeloid leukemia	Patients	Imetelstat	Promote lipid peroxidation and act as a ferroptosis inducer	Inhibit tumor metastasis and growth	Partial benefit	Phase II clinical trial	[305]
	Malignant glioma, breast cancer	Animal model, patients	Sulfasalazine	System Xc^−^ inhibitors	Overcome drug resistance	Partial benefit	Phase II clinical trial	[57]
	Colorectal cancer and breast cancer	Animal model, Patients	Artesunate	Regulate iron metabolism/generate reactive oxygen species	Inhibit tumor growth and reduce tumor recurrence	Partial benefit	Phase II clinical trial	[306, 307, 308]

**The key aspects of preclinical and clinical studies targeting ferroptosis pathway in human disease**.

*Abbreviations*: ACSL4, Acyl‐CoA synthetase‐4; NRF2, nuclear factor erythroid 2‐related factor 2; ATF4, activating transcription factor 4; SLC7A11, solute carrier family 7 member 11; GPX4, glutathione peroxidase 4; AMPK, adenosine 5‘‐monophosphate (AMP)‐activated protein kinase.

### 4.2 Emerging Technologies: Nanotechnology for the Targeted Delivery of Ferroptosis Modulators

Compared with normal cells, cancer cells exhibit elevated baseline ROS levels, increasing their vulnerability to further oxidative stress in conjunction with antioxidant inhibition [309]. Ferroptosis, characterized by the accumulation of lipid peroxides, has garnered significant attention because of its potential to overcome tumor resistance to conventional therapies and suppress highly metastatic tumors. However, the clinical translation of ferroptosis inducers is limited by challenges such as poor targeting efficiency, low bioavailability, and systemic toxicity. Nanotechnology addresses these limitations by enabling targeted delivery, controlled release, and codelivery of multiple agents, thereby improving their efficacy against ferroptosis and reducing off‐target effects [310]. Nanocarriers leverage passive tumor accumulation through the enhanced permeability and retention effect and can be surface engineered to ensure active targeting, optimizing the pharmacokinetics of ferroptosis modulators [311, 312]. Current nanoplatforms modulate ferroptosis through three core mechanisms [313, 314], representative examples of which are summarized below.

*Disrupting iron homeostasis*. Iron‐dependent Fenton reactions are the core triggers of lipid peroxidation and ferroptosis. Nanocarrier‐based iron delivery strategies could selectively increase intracellular iron levels in tumor tissues to activate the Fenton reaction, avoiding the nonspecific iron toxicity of systemic iron delivery. Typical representative materials used in iron‐based nanosystems, including iron oxide nanoparticles and iron‐based metal–organic frameworks, can be degraded in the acidic tumor microenvironment to release iron ions and initiate lipid peroxidation [315, 316]. For example, Feng et al. developed a metal–polyphenol network for efficient Fe^2+^ delivery [317], whereas Zhang et al. designed transferrin‐coupled vesicles that increased intracellular Fe^2+^ levels tenfold [318, 319]. Other strategies include the enhancement of transferrin‐dependent iron uptake and the induction of autophagic ferritin degradation, both of which amplify Fenton‐driven ferroptosis [320, 321]. Overall, nanomaterial‐mediated exogenous iron supplementation and intracellular iron redox regulation are the most direct and efficient approaches for disrupting tumor iron homeostasis and triggering ferroptosis.

*Activating lipid peroxidation*. Liposomes, or polymer‐based nanoparticles, deliver pro‐oxidants such as erastin or RSL3 to directly initiate lipid peroxidation [322]. High‐density lipoprotein‐like nanoparticles (HDL NPs) achieve synergistic “metabolism–ferroptosis” cell death by downregulating the expression of TXNRD1 and upregulating the expression of ACSL4, bidirectionally amplifying lipid peroxidation signals [323]. Additionally, nanocarriers can deliver exogenous unsaturated fatty acids that would act as peroxidation substrates [324], while biomimetic MoS_2_ nanosheets have been shown to directly catalyze lipid peroxidation [325]. In summary, these nanoplatforms integrate exogenous drug delivery and intrinsic catalytic functions to sustain and amplify lipid peroxidation signals, overcoming the insufficient ferroptosis activation induced by free small‐molecule‐based drugs.

*Suppressing antioxidant defenses*. Encapsulating GPX4 inhibitors such as RSL3 into nanocarriers—or using the inherent properties of nanomaterials to inhibit GPX4 activity—reduces the clearance of lipid peroxides and promotes ferroptosis [326]. Manganese dioxide nanoparticles loaded with sorafenib, arginine‐based nanobubbles, and pH‐sensitive quercetin‐loaded core–shell nanoparticles promote GSH depletion and GPX4 inactivation [327, 328, 329]. In addition to directly inhibiting GPX4 inhibition, emerging nanosystems block GSH biosynthesis by targeting key synthetic enzymes (GLS and γ‐GCS). Typical composite nanoplatforms, such as telaglenastat‐loaded mesoporous silica nanoparticles and L‐BSO‐modified AuSi@FePB nanosystems, effectively inhibit GSH synthesis and mitigate cellular antioxidant capacity, resulting in the long‐term activation of ferroptosis [330, 331]. Compared with single‐pathway interventions, this multitarget regulatory strategy significantly improves the stability and persistence of ferroptosis.

In summary, nanotechnology enables the precise, multipathway regulation of ferroptosis, showing strong potential in oncology, neuroprotection, and anti‐inflammatory therapy. Nevertheless, the translational application of ferroptosis‐regulating nanomaterials is still limited by prominent challenges. Unresolved issues include the insufficient biosafety of certain nanomaterials, the lack of standardized large‐scale preparation protocols, and unclear long‐term in vivo metabolic fates and biological effects. Future research should focus on the development of intelligent–responsive nanoplatforms with high biosafety and tumor specificity. In addition, exploring the crosstalk between nanomaterial‐induced ferroptosis and other cell death pathways, as well as tumor immune responses, will allow the construction of a new generation of precise, efficient, and safe nanomedicine–ferroptosis combination therapeutic strategies, laying a solid foundation for its clinical transformation.

### 4.3 Biomarkers for Ferroptosis: From Histochemistry to Circulating Indicators

Accurate detection and assessment of ferroptosis are essential for elucidating its underlying pathophysiological mechanisms and developing targeted therapeutic strategies. To this end, the identification of ferroptosis biomarkers has evolved from direct morphological and compositional observations based on histochemistry to the discovery of stable, quantifiable circulating indicators in body fluids.

*Histochemical classical markers*. Histochemical methods enable the direct confirmation of ferroptosis at the tissue or cellular level and thus serve as a foundation for mechanistic research and early diagnosis. Key classical markers include those reflecting mitochondrial ultrastructural alterations, iron accumulation, lipid peroxidation products, and the differential expression of critical genes or proteins. Characteristic mitochondrial changes in ferroptosis—such as reduced volume, diminished or absent cristae, outer membrane rupture, and increased membrane density—can typically be observed with transmission electron microscopy [332]. However, these morphological features are relatively nonspecific, as they may also occur under conditions of oxidative stress or general mitochondrial dysfunction. Iron deposition, which involves the accumulation of Fe^2+^ and Fe^3+^ in tissues such as the heart, liver, spleen, and kidney, is positively associated with iron overload and a prerequisite for ferroptosis. Detection techniques include Prussian blue staining and fluorescent iron ion probes [333]. Lipid peroxidation products—including oxidized PUFA‐PE derivatives and oxidation byproducts such as 4‐HNE, MDA, and 8‐hydroxy‐2′‐deoxyguanosine—are considered specific indicators of ferroptosis [163, 334]. Common approaches for detecting lipid peroxidation include fluorescent imaging with the BODIPY 581/591 C11 probe, lipidomics analysis, thiobarbituric acid reactive substances (TBARS) assays, and immunohistochemical staining for MDA and 4‐HNE. Among these methods, flow cytometry following BODIPY 581/591 C11 staining is highly sensitive and reliable for assessing lipid peroxidation. Immunohistochemistry‐ or immunofluorescence‐detected alterations in key gene or protein expression levels—such as downregulation or loss of GPX4 and SLC7A11/xCT expression and upregulation of CHAC1, ACSL4, PTGS2, NOX1, and SOD expression—can reflect a ferroptosis‐susceptible phenotype. The downregulation of FTH1 expression indicates the depletion of ferritin stores, whereas the translocation of TFR1 from the Golgi apparatus to the plasma membrane is considered pivotal for the initiation of ferroptosis [335]. Additionally, hyperoxidized peroxiredoxin 3 (PRDX3) has been identified as a novel and specific marker of ferroptosis [336]; unlike in those treated with inducers of other forms of cell death or nonmitochondrial oxidative stress, hyperoxidized PRDX3 is specifically detected in cells treated with inducers of ferroptosis such as erastin and RSL3. However, its occurrence and clinical relevance in human tissue samples, particularly tumor specimens, remain to be fully established.

*Circulating biomarkers*. Biomarkers present in circulating bodily fluids such as blood and cerebrospinal fluid offer several advantages, including noninvasiveness, potential for dynamic monitoring, and feasibility for clinical translation, making them an important area of current research. MDA, 4‐HNE, and hexanoyl‐lysine (HEL) adducts are among the most commonly measured end products of lipid peroxidation in circulation. In a MS cohort, plasma 4‐HNE concentrations were significantly lower in patients with progressive MS than in those with relapsing‐remitting MS and were associated with disease progression and fatigue scores, suggesting a potential role in differentiating disease phenotypes [337]. Oxidized phosphatidylethanolamines and phosphatidylcholines can be sensitively detected in plasma or serum using liquid chromatography‒tandem mass spectrometry (LC‒MS/MS), providing direct molecular evidence of lipid peroxidation with relatively high specificity [338]. Serum levels of free iron, transferrin, and ferritin reflect the size of the systemic iron pool and iron use status [337]. Measurement of the plasma GSH/GSSG ratio, as well as GPX4 activity or expression levels, may help predict tumor cell sensitivity to ferroptosis‐inducing agents targeting GPX4, such as altretamine and sorafenib [339]. In addition to conventional proteins and metabolites, circulating exosomal microRNAs have been explored for monitoring ferroptosis. For instance, the levels of miR‐375‐3p in plasma exosomes from patients with Stevens‐Johnson syndrome/toxic epidermal necrolysis (SJS/TEN) are positively correlated with intracellular Fe^2+^ and MDA concentrations but negatively correlated with the concentrations of coenzyme Q10, suggesting the potential of miR‐375‐3p as a noninvasive biomarker of ferroptosis activity in the context of severe adverse cutaneous reactions [340]. Furthermore, ACSL4 and GPX4 exhibit significant diagnostic and prognostic value in sepsis, including the ability to predict 28‐day mortality, and may serve as novel serum markers for sepsis diagnosis and risk stratification [341]. In summary, biomarkers play pivotal roles in ferroptosis research, offering insights into disease mechanisms, prognoses, treatment responses, and drug development.

### 4.4 The Challenge of Context‐Dependent Therapy and Therapeutic Window

Ferroptosis is a distinct form of RCD driven by iron‐dependent lipid peroxidation and tightly regulated through multiple molecular mechanisms. Targeting ferroptosis holds significant therapeutic potential in various diseases, including cancer, cardiovascular disorders, and neurodegenerative conditions. Despite its promising prospects, ferroptosis research faces several critical challenges. First, cellular heterogeneity and differential sensitivity are prominent: Different cell types exhibit varying susceptibility to ferroptosis, and even within the same tumor, cancer cells may respond differently due to genetic background, metabolic state, or microenvironmental influences. For example, certain cancer cells resist ferroptosis by relying on antioxidant defense systems such as the GSH–GPX4 axis or the FSP1–CoQ_10_ pathway, whereas others are more vulnerable owing to dysregulated iron homeostasis or abnormal lipid composition. To solve this problem, stratified and cell‐type‐specific intervention strategies are the core solution. Future research needs to conduct multiomics profiling (genomics, lipidomics, and transcriptomics) to classify cell subgroups with distinct ferroptosis vulnerability. For cancer cells relying on the GSH–GPX4 or FSP1–CoQ10 antioxidant pathways to develop ferroptosis resistance, combined inhibitors targeting these parallel defensive axes can be adopted to block compensatory mechanisms. For cells characterized by iron overload and excessive PUFA‐PLs that are inherently sensitive to ferroptosis, low‐dose targeted inducers can be applied to exert therapeutic effects. In addition, targeting stromal cells and immune cells within the TME to cut off the metabolic support for tumor cells is also a viable direction, which can reverse the microenvironment‐mediated ferroptosis resistance. Second, there is a substantial risk of toxicity to normal tissues, as key regulatory components of ferroptosis—such as GPX4 and System Xc^−^—are also essential for normal cellular function. Systemic inhibition of these pathways may induce ferroptosis in vital organs (liver, kidneys, and nervous system), leading to severe adverse effects. For instance, GPX4 knockout in mice results in embryonic lethality or organ failure in adults, which limits the clinical dosing range and applicability of ferroptosis inducers [342]. For normal tissue toxicity and narrow therapeutic window, precision delivery and stage‐specific intervention strategies are prioritized. Advanced biomaterial delivery systems are developed to solve this problem: functionalized nanoparticles, liposomes, and hydrogels equipped with tumor‐specific ligands, pH‐responsive or ROS‐responsive release structures can achieve localized drug release, reduce systemic exposure, and protect normal tissues. Furthermore, disease‐stage differentiated therapy is advocated: for acute injury such as myocardial I/R damage, ferroptosis inhibitors are used in the early stage to relieve tissue loss; for tumor treatment, phased application of ferroptosis inducers avoids sustained damage to normal tissues. Third, the lack of specific and reliable biomarkers remains a major obstacle. Currently, no validated molecular markers exist for accurately detecting ferroptosis events or predicting tumor cell sensitivity to ferroptosis inducers. Conventional detection methods, such as measurements of lipid peroxidation or rescue experiments using ferroptosis inhibitors, often fail to clearly distinguish ferroptosis from other forms of cell death. Emerging research suggests that overcoming the biomarker challenge requires a shift from reliance on single markers to integrated multimodal detection platforms. A comprehensive approach that combines intracellular and extracellular markers may offer a more robust solution for diagnosing and monitoring ferroptotic damage. For instance, the simultaneous measurement of lipid peroxidation products (e.g., malondialdehyde, 4‐hydroxynonenal) alongside iron distribution patterns and the presence of specific degradation intermediates—such as ferritin fragments released during ferritinophagy—could provide a more accurate signature of ferroptotic activity. Furthermore, the use of advanced imaging techniques, including mass spectrometry imaging and fluorescent probes targeting lipid peroxidation or labile iron pools, could enable spatial mapping of ferroptotic events within tissues.

Regarding the therapeutic time window, three key issues must be addressed. (1) Dosage and timing must be precisely balanced: the efficacy of ferroptosis inducers depends on optimal dosing and exposure duration. Subtherapeutic doses may fail to trigger ferroptosis, while excessive doses can damage normal cells or induce immunosuppression. Thus, individualized treatment regimens should be determined based on tumor sensitivity, patient physiology, and drug metabolism. (2) Dynamic changes in the tumor microenvironment—such as hypoxia, acidosis, and inflammatory signaling—can modulate ferroptosis induction. During therapy, cancer cells may adapt by upregulating antioxidant genes or reprogramming lipid metabolism, thereby evading cell death and shortening the effective treatment window. Real‐time monitoring of the microenvironment is therefore essential to enable timely intervention. (3) The integration with conventional therapies presents synergistic and sequencing challenges. Ferroptosis inducers are frequently combined with chemotherapy, radiotherapy, or immunotherapy. While some chemotherapeutic agents enhance ferroptosis sensitivity via DNA damage or oxidative stress, and radiotherapy promotes lipid peroxidation through free radical generation, the optimal sequence and dosage ratios require further refinement. In conclusion, ferroptosis research stands at a pivotal juncture in transitioning from basic discovery to clinical translation. Overcoming existing barriers—particularly achieving precise targeting and dynamically optimizing the therapeutic window—is central to future progress. Emerging approaches, including nanotechnology‐based delivery, combination strategies (e.g., ferroptosis–immunotherapy crosstalk), and artificial intelligence (AI)‐driven patient stratification, are actively being explored to address these challenges. Combination therapy with conventional clinical regimens is widely verified. Ferroptosis inducers are combined with chemotherapy, radiotherapy, and immunotherapy: chemotherapeutics and radiotherapy can amplify lipid peroxidation and boost ferroptosis sensitivity, while ferroptotic tumor cells release DAMPs to activate antitumor immunity, forming a synergistic effect. Further exploration on the optimal administration sequence and dosage ratio of combined drugs will help to maximize efficacy. Second, nanomedicine platforms are vigorously developed. Smart nanocarriers can codeliver multiple drugs to simultaneously interfere with iron metabolism, GSH synthesis, and GPX4 activity and realize spatiotemporal controllable drug release, which greatly expands the therapeutic window. Third, AI technology is introduced into the whole treatment process. AI algorithms analyze multiomics data, imaging results and clinical indicators to achieve intelligent patient stratification, predict tumor ferroptosis susceptibility and screen suitable therapeutic regimens, providing data support for personalized treatment. Fourth, natural compounds and repurposed drugs are excavated. A series of natural extracts and clinically approved drugs have been found to regulate ferroptosis with favorable safety profiles, which shortens the drug development cycle and lowers clinical transformation risks.

In conclusion, overcoming the barriers to ferroptosis‐based therapy requires a paradigm shift from generic induction or inhibition to precision ferroptosis modulation. This entails biomarker‐guided patient selection, context‐specific targeting of resistance nodes, advanced delivery systems that separate efficacy from toxicity, and adaptive dosing informed by dynamic monitoring. Future research should prioritize the validation of multianalyte biomarker panels, the development of tumor‐selective prodrugs and nanocarriers, and the execution of adaptive clinical trials that incorporate real‐time ferroptosis assessment. By systematically addressing these challenges, ferroptosis‐targeted therapies can be safely and effectively translated into clinical practice for cancer, neurodegeneration, and beyond.

## 5 Conclusions and Future Perspectives

Ferroptosis is a novel form of RCD driven by iron‐dependent lipid peroxidation and has become a major research hotspot in life sciences and biomedicine. Distinct from conventional cell death modalities, the fate of cells undergoing ferroptosis is governed by the dynamic balance of three regulatory networks: antioxidant defense, iron metabolism, and lipid metabolism, endowing it with unique mechanisms and vital biological functions. Aberrant activation of ferroptosis is closely linked to the pathogenesis of multiple major human diseases, including tumors, cardiovascular disorders, neurodegenerative diseases, and acute organ injury. Owing to its discovery, universal distribution, and key pathogenic function in human diseases, ferroptosis has emerged as a highly potential broad‐spectrum therapeutic target. Currently, researchers worldwide are focusing on the regulatory mechanisms of ferroptosis and exploring pharmacological interventions and genetic modulation strategies. Precise regulation of ferroptosis is expected to achieve targeted therapy for multiple major diseases and provide novel strategies for clinical diagnosis and treatment.

The discovery of ferroptosis has opened up a new platform for the study of human diseases. However, several challenges persist: (1) lipid peroxidation and iron dysregulation are not exclusive to ferroptosis and can occur in other cell death pathways such as apoptosis and necrosis. Moreover, (2) a lack of ferroptosis‐specific biomarkers exists. Although elevated TFR1 may help distinguish between ferroptosis and apoptosis [335], TFR1 is widely expressed and regulated by various factors, including intracellular iron homeostasis. Recently, PRDX3 has been considered as a potential marker of ferroptosis [336]. However, cells lacking PRDX3 can still undergo ferroptosis. Currently, lipid peroxidation is the most widely used biomarker of ferroptosis, but lipid peroxidation does not necessarily lead to cell death because a critical threshold of LOOH must be reached to trigger ferroptosis [343]. Furthermore, the mechanisms by which lipid peroxidation propagates within cells, as well as the differential susceptibility of various lipid classes to peroxidative degradation, remain critical questions that require further investigation. (3) Ferroptosis‐based drug development and delivery face difficulties. How to precisely deliver drugs to target tissues or cells (such as tumor tissue, neurons in specific brain regions) while avoiding damage to normal tissues is a major challenge. In addition, most existing ferroptosis inducers or inhibitors suffer from problems such as low selectivity, poor pharmacokinetic properties, potential off‐target effects, and toxicity. Consequently, the development of precise, targeted therapeutic strategies that selectively induce ferroptosis in malignant cells is essential to maximize the efficacy and safety of such treatments.

Future research on ferroptosis is expected to advance along multiple frontiers, with a core focus on precisely distinguishing the characteristic differences between ferroptosis and other forms of cell death. A deeper understanding is needed of the specific regulatory mechanisms underlying lipid peroxidation and iron metabolism dysregulation across various cell death pathways in different cell types and disease contexts, thereby clarifying the unique molecular patterns and regulatory rules governing ferroptosis. concurrently, the discovery, screening, and rigorous validation of specific and exclusive biomarkers for ferroptosis represent a critically valuable research direction, aiming to address the current challenge of lacking reliable biomarkers. Such biomarkers would enable precise discrimination of ferroptosis from other cell death modalities and facilitate disease diagnosis, treatment response evaluation, and patient prognosis. Furthermore, overcoming the numerous technical hurdles in the development and delivery of ferroptosis‐targeting drugs is another key trend. By leveraging cutting‐edge biomedical technologies to optimize drug discovery pipelines and innovate drug delivery strategies, researchers aim to develop highly efficient, targeted ferroptosis‐modulating agents. Combining these with individual patient pathophysiological profiles will enable the design of safe and effective precision therapies, thereby breaking through existing bottlenecks in clinical translation.

Specifically, the following directions warrant particular attention: first, precise cell death typing. To overcome the challenge of mixed cell death and single‐marker limitations, integrate single‐cell genomics, spatial transcriptomics, proteomics, metabolomics, lipidomics, redox ratios, and core gene expression signatures to accurately distinguish ferroptosis from other cell death pathways. Second, mechanism research at the clinical level. Move beyond the classical GPX4–glutathione axis; elucidate non‐cell‐autonomous signaling, intercellular communication, and oncogenic metabolic reprogramming in ferroptosis. Incorporate NF2–YAP signaling and KRAS‐driven mitochondrial remodeling to establish pathologically relevant disease models for evaluating metabolic‐ and microenvironment‐targeted interventions. Third, cell‐specific drug development. Address the lack of cellular specificity by profiling differences in lipid composition and mitochondrial metabolism among tumor, normal, and immune cells. Leverage cancer‐enriched PUFAs and mitochondrial dependency to design precise ferroptosis inducers for targeted killing of diseased cells. Finally, combination therapy. Combine ferroptosis targeting with metabolic inhibitors (DHODH, glycolysis, glutamine metabolism), nanomedicine, and conventional therapies to synergistically enhance antitumor efficacy and overcome chemoresistance, while refining mechanistic and safety assessments.

Thus, as an emerging paradigm in RCD, ferroptosis offers novel insights into the pathogenesis of major diseases and opens innovative avenues for therapeutic development. With ongoing advancements in mechanistic understanding and technological capabilities, ferroptosis‐targeted interventions are poised to become a cornerstone of precision medicine.

## Author Contributions

All the authors contributed significantly to this work. Yuxia Zhou and Dandan Xiao provided direction and guidance throughout the preparation of this manuscript. Dandan Xiao, Lin Ye, Tianqi Jiang, and Mengyuan Liu collected and prepared the related literature. Dandan Xiao and Lin Ye drafted the manuscript. Yuxia Zhou reviewed and made significant revisions to the manuscript. All authors have read and approved the final manuscript.

## Conflicts of Interest

The authors declare no conflicts of interest.

## Funding

This work was supported by Natural Science Foundation of Shandong Province (ZR2025QC353) and Medical and Health Science and Technology Project of Shandong Province (202311000985).

## Ethics Statement

The authors have nothing to report.

## Data Availability

The authors have nothing to report.
